# Subtitled speech: the neural mechanisms of ticker-tape synaesthesia

**DOI:** 10.1093/brain/awae114

**Published:** 2024-04-15

**Authors:** Fabien Hauw, Benoît Béranger, Laurent Cohen

**Affiliations:** Inserm U 1127, CNRS UMR 7225, Sorbonne Universités, Institut du Cerveau, ICM, Paris 75013, France; AP-HP, Hôpital de La Pitié Salpêtrière, Fédération de Neurologie, Paris 75013, France; Inserm U 1127, CNRS UMR 7225, Sorbonne Universités, Institut du Cerveau, ICM, Paris 75013, France; Inserm U 1127, CNRS UMR 7225, Sorbonne Universités, Institut du Cerveau, ICM, Paris 75013, France; AP-HP, Hôpital de La Pitié Salpêtrière, Fédération de Neurologie, Paris 75013, France

**Keywords:** synaesthesia, ticker-tape synaesthesia, reading, functional MRI

## Abstract

The acquisition of reading modifies areas of the brain associated with vision and with language, in addition to their connections. These changes enable reciprocal translation between orthography and the sounds and meaning of words. Individual variability in the pre-existing cerebral substrate contributes to the range of eventual reading abilities, extending to atypical developmental patterns, including dyslexia and reading-related synaesthesias. The present study is devoted to the little-studied but highly informative ticker-tape synaesthesia, in which speech perception triggers the vivid and irrepressible perception of words in their written form in the mind’s eye.

We scanned a group of 17 synaesthetes and 17 matched controls with functional MRI, while they listened to spoken sentences, words, numbers or pseudowords (Experiment 1), viewed images and written words (Experiment 2) or were at rest (Experiment 3).

First, we found direct correlates of the ticker-tape synaesthesia phenomenon: during speech perception, as ticker-tape synaesthesia was active, synaesthetes showed over-activation of left perisylvian regions supporting phonology and of the occipitotemporal visual word form area, where orthography is represented. Second, we provided support to the hypothesis that ticker-tape synaesthesia results from atypical relationships between spoken and written language processing: the ticker-tape synaesthesia-related regions overlap closely with cortices activated during reading, and the overlap of speech-related and reading-related areas is larger in synaesthetes than in controls. Furthermore, the regions over-activated in ticker-tape synaesthesia overlap with regions under-activated in dyslexia. Third, during the resting state (i.e. in the absence of current ticker-tape synaesthesia), synaesthetes showed increased functional connectivity between left prefrontal and bilateral occipital regions. This pattern might reflect a lowered threshold for conscious access to visual mental contents and might imply a non-specific predisposition to all synaesthesias with a visual content.

These data provide a rich and coherent account of ticker-tape synaesthesia as a non-detrimental developmental condition created by the interaction of reading acquisition with an atypical cerebral substrate.

## Teaser

When hearing speech, ticker-tape synaesthetes automatically and vividly perceive the words in written form in their mind's eye. Using task-related and resting state functional MRI, Hauw *et al.* study 17 affected individuals and reveal the atypical functioning of their reading system, including regions also implicated in dyslexia.

## Introduction

The acquisition of reading leads to deep changes in brain areas related to vision and language and in their connections,^1^ enabling the translation from orthography to sound and meaning during reading and the opposite translation during spelling. Key among those changes is the emergence of a small area sharply specialized for recognition of letters in the left fusiform gyrus, a region known as the visual word form area (VWFA).^2,3^ The VWFA has privileged connections with language areas in the left perisylvian cortex.^4^ This enhanced anatomical and functional connectivity exists before reading acquisition^5,6^ and is reinforced by reading acquisition.^7,8^

All changes associated with reading acquisition act on a pre-existing cerebral substrate whose individual features, determined genetically in part, modulate the acquisition of reading and contribute to the eventual variability in reading skills. Such variability includes the range of typical reading, as shown by a host of twin studies and genome-wide association studies.^9-11^ In a minority of the population, atypical brain organization is revealed through dyslexia, a variety of selective impairments in learning to read,^12^ associated with abnormal patterns of brain activation^13^ and connectivity.^14^

In another few per cent of children, atypical brain organization does not yield reading impairment but unusual perceptual contents, or synaesthesias, associated with written language.^15,16^ The present study is devoted to the little-studied but highly informative ticker-tape synaesthesia (TTS), a condition first described by Francis Galton,^17^ in which speech perception triggers the irrepressible and vivid perception of words in their written form in the mind’s eye.^18-20^ The most studied synaesthesia involving letters is grapheme-colour synaesthesia, in which each letter is perceived in association with a given colour (e.g. Hubbard and Ramachandran^21^), but other synaesthesias have also been reported, including the attribution to letters of well-defined sex-related and personality traits (see www.thesynesthesiatree.com).

TTS is the only synaesthesia in which both the ‘inducer’ and the ‘concurrent’ are in the realm of language, with phonological and orthographic representations, respectively. Indeed, auditory stimuli trigger TTS inasmuch as they can be coded phonologically: real words with regular or irregular orthography, pseudowords, unfamiliar languages with a familiar phonology, and even natural sounds with a conventional onomatopoeic transcription.^18^ This implies that the strings of letters that materialize in the mental imagery of synaesthetes are derived from speech through the two usual routes that operate during reading: a phonological route implementing phoneme–grapheme correspondences and a lexical route retrieving stored word spelling.^18,22^ We therefore propose that TTS reflects exceptionally powerful and automatic top-down influences from perisylvian speech areas, in which phonology is processed, onto the VWFA, which supports orthographic representations. This theory gained initial support in the recent study of a single case of a ticker-tape synaesthete.^23^ Our main findings were that, in comparison to controls, when this individual was listening to speech there was an over-activation of perisylvian cortices and of the VWFA, a set of regions also involved in reading. In the present study, we aim to assess the generality and robustness of those early findings by studying a larger sample of synaesthetes and to extend its scope by studying functional connectivity, a key player in the pathophysiology of synaesthesia.

A group of 17 ticker-tape synaesthetes and matched controls were scanned with functional MRI while they listened to speech (Experiment 1), viewed written words and other images (Experiment 2) or were at rest (Experiment 3). This protocol was designed to assess the following set of predictions.

Given that TTS consists of the vivid perception of letters generated from sound, our first prediction was that speech perception should activate perisylvian language areas involved in speech processing and phonology-to-orthography translation, and ventral occipito-temporal regions subtending orthography, i.e. the VWFA, more strongly than they do in typical individuals.^24^ Given that we observed previously that TTS was triggered by any auditory input provided that it has phonological content, our second prediction was that TTS-related activations should occur equally while synaesthetes are listening to real or pseudowords.^18^ Thus, considering TTS as a kind of upended reading process, i.e. running from sounds to letters rather than the opposite, our third prediction was that regions involved in TTS should overlap with those associated with reading and dyslexia. Finally, our fourth and less specific expectation was based on the common hypothesis that synaesthesias result from increased links between brain regions processing the inducer and the concurrent.^25,26^ Hence, we predicted that the pattern of functional connectivity during rest might reveal underlying predispositions to TTS, measurable even in the absence of speech perception. Assuming that all synaesthesias with a visual component result from an easier access to consciousness of internally generated images, we also predicted that some predisposition to TTS should be shared with other types of associated synaesthesia.

## Materials and methods

### Participants

We included 17 TTS participants and 17 controls, all right-handed native French speakers, matched for age, biological sex and education, with no history of neurological or psychiatric disorders. Synaesthetes experienced TTS whenever listening to speech and enjoyed an objective advantage over controls in behavioural tasks.^20^ Full details on participants are provided in the Supplementary material. The research was approved by the institutional review board of the INSERM (protocol C13-41), and all participants provided informed written consent in accordance with the Declaration of Helsinki.

### MRI experiments

The full information on MRI experiments is reported as Supplementary methods and summarized in the ‘Results' section.

## Results

### Experiment 1: functional MRI study of speech perception

The aim of this experiment was to compare brain activation during conditions eliciting TTS and in control conditions. Participants were presented with an alternation of short auditory blocks of: sentences; lists of words, pseudowords or numbers; scrambled sentences; and rest, while they had to detect an oddball stimulus (the spoken non-word, ‘tatatata’).

#### Behavioural performance

There was no significant difference between groups on the detection task, for the hit rate [89.4% in synaesthetes versus 95.6% in controls, *t* = −1.39, *P* = 0.17, 95% confidence interval (CI) (−3.05 to 0.58)] nor for the number of false alarms (mean = 0.06 in both synaesthetes and controls).

#### Activation by speech

**Figure 1:**
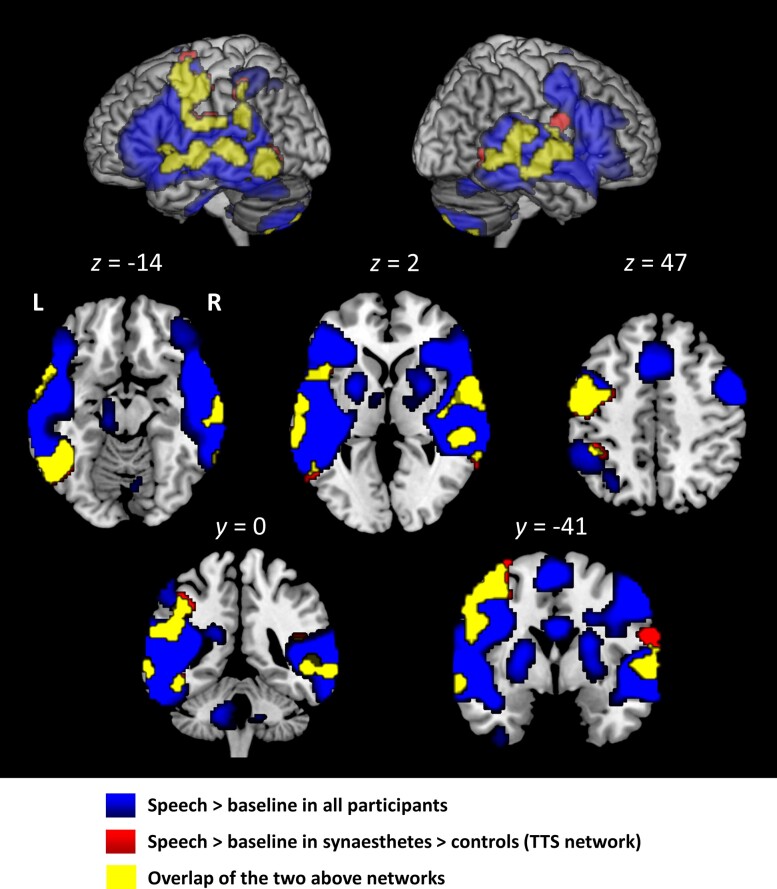
**Experiment 1.** Activation by speech > baseline in all participants (blue), in synaesthetes more than in controls (red), and the overlap of those two volumes (yellow). A set of left-predominant regions, the ‘TTS network’, in the frontal, parietal and temporal lobes, were more activated in synaesthetes than in controls. Abbreviation: TTS = ticker-tape synaesthesia.

**Figure 2:**
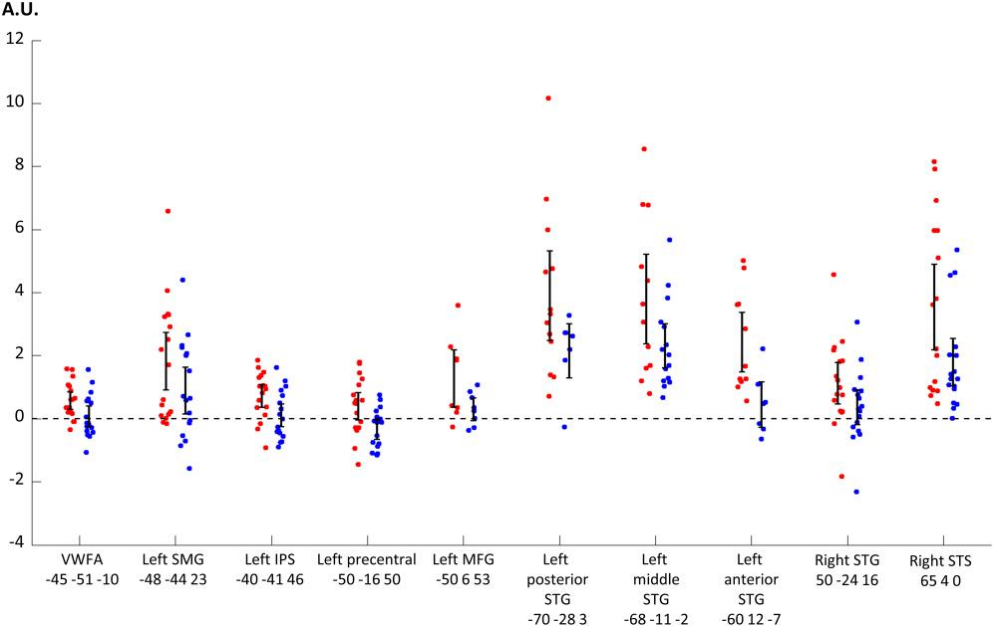
**Individual activations in the TTS network.** In Experiment 1, we compared brain activation for speech > baseline in synaesthetes > controls and identified a set of regions that we termed the ‘TTS network’. In the group-level peak voxel of each region, we plotted individual contrast estimates of the contrast speech > baseline, in synaesthetes (red dots) and controls (blue dots). The 95% CIs of groups are represented as black bars. In all regions, synaesthetes had a higher mean activation than controls. A few values are missing, because the group-level peak coordinates might occasionally fall outside the masks in which individual statistics were computed. Activation is plotted in arbitrary units (A.U.). Abbreviations: IPS = intraparietal sulcus; MFG = middle frontal gyrus; SMG = supra-marginal gyrus; STG = superior temporal gyrus; TTS = ticker-tape synaesthesia; VWFA = visual word form area.

Initially, we averaged all conditions triggering TTS, i.e. all conditions except scrambled sentences, and compared them with baseline (Fig. 1 and Supplementary Fig. 1 for Hedges’ *g* effect size maps). Averaging across both groups, speech activated a broad left-predominant fronto-parieto-temporal network covering the usual language areas and extending ventrally to the fusiform gyrus in the left hemisphere. We then contrasted synaesthetes minus controls [voxelwise *P* < 0.001 and clusterwise *P* < 0.05 family-wise error (FWE) corrected; Table 1], masking by activation in synaesthetes relative to baseline (voxelwise *P* < 0.001). Synaesthetes had stronger activation: (i) in the left-hemispheric ventral occipito-temporal/VWFA [Montreal Neurological Institute (MNI) coordinates −45, −51, −10, *Z* = 6.22], supra-marginal gyrus (SMG; MNI −48, −44, 23, *Z* = 5.30) extending to the intraparietal sulcus (IPS; MNI −40, −41, 46, *Z* = 4.50), and precentral cortex (MNI −50, −16, 50, *Z* = 5.92) extending to the posterior middle frontal gyrus (MNI −50, 6, 53; *Z* = 5.56); and (ii) in the bilateral superior temporal gyrus/sulcus (STG/STS) (left: posterior, MNI −70, −28, 3, *Z* > 8; middle, MNI −68, −11, −2, *Z* = 5.99; anterior, MNI −60, 12, −7, *Z* = 7.59; right: MNI 50, −24, 16, *Z* = 6.67; MNI 65, 4, 0, *Z* = 7.43). Plots of data for individuals at the coordinates of the peak of each cluster show that in each region, synaesthetes had a higher mean activation than controls (Fig. 2), and we also report the 95% CI of contrast estimate from the ANOVA, which never included zero (Table 1). For simplicity, this set of areas will be referred to as ‘the TTS network’. The converse contrast of controls minus synaesthetes, masked by the activation in controls (voxelwise *P* < 0.001), showed no stronger activation in controls than in synaesthetes.

**Table 1:** Experiment 1: regions with higher activation in synaesthetes than in controls for the contrast speech > baseline

	MNI coordinates			Group difference		Effect size	
	*x*	*y*	*z*	*Z*-value	95% CI	Hedges’ *g*	95% CI
VWFA	45	−51	−10	6.22	0.20–0.37	3.78	2.59–4.99
Left SMG	−48	−44	23	5.3	0.24–0.51	3.16	2–4.34
Left IPS	−40	−41	46	4.5	0.21–0.51	2.64	1.5–3.8
Left precentral	−50	−16	50	5.92	0.32–0.60	3.57	2.4–4.78
Left MFG	−50	6	53	5.56	0.19–0.38	3.33	2.16–4.52
Left posterior STG	−70	−28	3	>8	0.55–0.82	5.62	4.34–6.95
Left middle STG	−68	−11	−2	5.99	0.31–0.59	3.62	2.44–4.83
Left anterior STG	−60	12	−7	7.59	0.35–0.55	4.8	3.57–6.08
Right posterior STG	50	−24	16	6.67	0.34–0.59	4.11	2.91–5.34
Right anterior STG	65	4	0	7.43	0.44–0.71	4.67	3.45–5.94

For each region, the table indicates the MNI coordinates of the peak voxel, the *Z*-value and the 95% confidence interval (CI) of the group difference in this voxel, the associated Hedges’ *g* effect size and its 95% CI.

Abbreviations: IPS = intraparietal sulcus; MFG = middle frontal gyrus; SMG = supra-marginal gyrus; STG = superior temporal gyrus; VWFA = visual word form area.

#### Comparisons between types of speech

We then made comparisons among the four speech conditions: words versus pseudowords, words versus sentences and words versus numbers. We used univariate contrasts and multivariate pattern analysis decoding. Averaging both groups, as expected, we found significant differences between conditions (Supplementary Figs 2 and 3). In particular, there was significant decoding of words from pseudowords in a temporoparietal region overlapping with both the SMG and the posterior MTG components of the TTS network (Supplementary Fig. 3, bottom panel). Importantly, for all those analyses, we found no significant differences between synaesthetes and controls. This indicates that apart from synaesthesia, speech processing did not differ between the two groups.

#### Contrasting sentences and scrambled sentences

We then assessed the minimal contrast of sentences minus scrambled sentences. Averaging both groups, we found an extensive bilateral fronto-parieto-temporal network covering language areas. We contrasted synaesthetes minus controls (voxelwise *P* < 0.001 and clusterwise *P* < 0.05 FWE corrected), masking by activation in synaesthetes (voxelwise *P* < 0.001). We found no significant difference between groups. There was still, somewhat below the threshold for cluster extent, a left SMG cluster [MNI −58, −41, 23, *Z* = 4.36; Hedges’ effect size *g* = 2.19, 95% CI (1.21–3.18); 99 voxels] and a right STS cluster [MNI 50, −34, 3, *Z* = 4.22; *g* = 2.11, 95% CI (1.14–3.11); 103 voxels]. We hypothesized that the lack of significance of those clusters did not result from their weakness but from their intrinsically small size. This was confirmed by performing the group-level analysis without smoothing individual images, hence reducing spatial correlation and shrinking the cluster-size threshold. Both clusters then reached significance [left SMG: MNI −60, −41, 23, *Z* = 4.78; *g* = 2.42, 95% CI (1.43–3.42); right STS: MNI 50, −34, 3, *Z* = 5.01; *g* = 2.54, 95% CI (1.55–3.56)]. The converse contrast of controls minus synaesthetes, masked by the activation in controls (voxelwise *P* < 0.001), showed no stronger activation in controls than in synaesthetes.

Considering that the lack of significance could also result from individual variability in the location of small activation foci, we extracted the average contrast value in the 10% best voxels of each participant, in 10-mm-radius spheres centred on the six main left-hemispheric peaks of the contrast of sentences minus scrambled sentences averaged over both groups. The sentences > scrambled difference was larger in synaesthetes than in controls in the left SMG [*t*(32) = 2.20, *P* = 0.035] and inferior frontal gyrus [*t*(32) = 2.85, *P* = 0.008].

#### Overlap of the ticker-tape synaesthesia network with regions involved in dyslexia

TTS and dyslexia both result from atypical development of the cerebral reading system. To determine whether common areas are involved in both cases, we referred to four meta-analyses of imaging studies, showing reproducible areas of reduced activation in dyslexia.^27-30^ Following the method of Feng *et al*.,^13^ we collected 30 left-hemispheric coordinates from those meta-analyses and averaged nearby coordinates to create 10 spherical regions of interest (ROIs) of radius 8 mm (Supplementary Table 1). There was a striking overlap between those ROIs and the TTS network (Supplementary Fig. 4). Within each ROI, we assessed whether the average activation by speech > baseline was larger in synaesthetes than in controls. This was the case for the inferior temporal gyrus and fusiform ROIs close to the usual peak of the VWFA^31^ [MNI −50, −60, −9, false discovery rate (FDR)-corrected *P* < 0.001; MNI −44, −46, −18, FDR-corrected *P* = 0.003], for nearby SMG and posterior STG ROIs (MNI −49, −44, 35, FDR-corrected *P* = 0.015; MNI −52, −43, 21, FDR-corrected *P* < 0.001) and for the posterior MTG ROI (MNI −54, −51, 5, FDR-corrected *P* = 0.035). Note that even non-significant ROIs were partly overlapping with or contiguous with the TTS network.

#### Psychophysiological interaction

**Figure 3:**
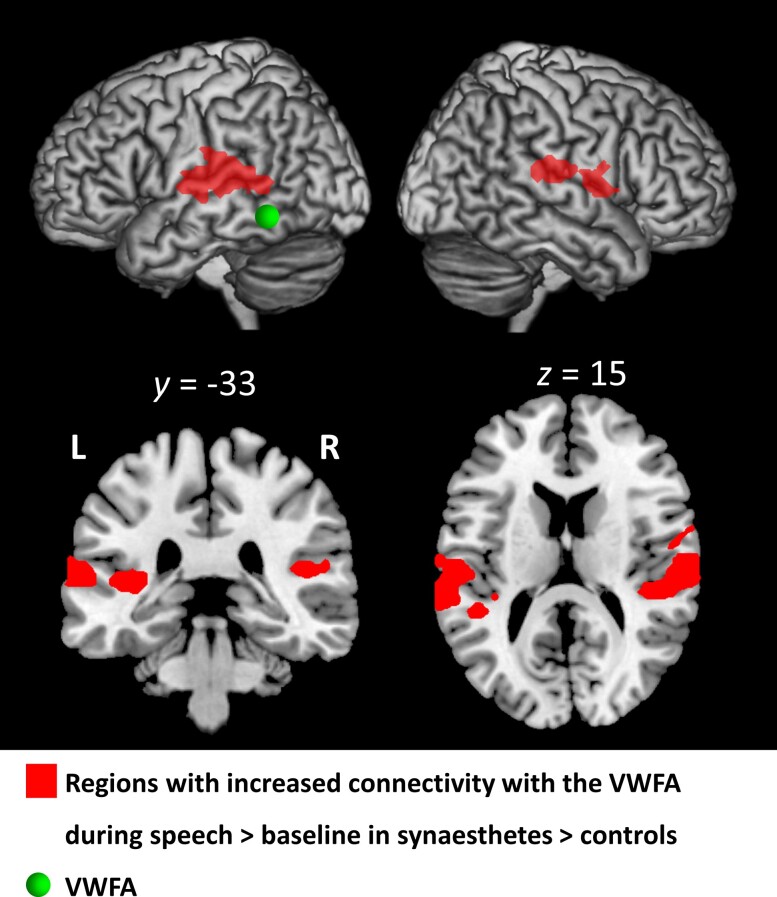
**Experiment 1.** Psychophysiological interaction analysis of the TTS network. During speech perception, the visual word form area (VWFA; centred on the green sphere) showed increased connectivity with left-predominant posterior STG/SMG cortex (red). Abbreviations: L = left; R = right; TTS = ticker-tape synaesthesia.

We then moved to analyses of functional connectivity. We reasoned that, during speech perception in synaesthetes, the TTS network might have not only abnormally intense activation, as shown before, but also abnormally high connectivity, intrinsically or with other regions. We used psychophysiological interaction to identify such regions: for each of the nine ROIs of the TTS network, we looked for voxels whose increase in connectivity with the ROI during speech perception versus baseline would be larger in synaesthetes than in controls. To limit the analysis to areas activated by speech, the analysis was masked with the volume activated by speech perception minus the baseline in synaesthetes (voxelwise *P* < 0.001). Only the VWFA showed increased speech-dependent connectivity in synaesthetes, specifically with the bilateral posterior STG/SMG [Fig. 3; left: MNI −68, −36, 13, *Z* = 4.33; *g* = 1.46, 95% CI (0.80–2.13); right: MNI 65, 6, 8, *Z* = 4.79; *g* = 1.62, 95% CI (0.96–2.29); MNI 65, −18, 20, *Z* = 4.78; *g* = 1.62, 95% CI (0.96–2.29)]. Using a similar masking by the volume activated by speech perception minus baseline in controls (voxelwise *P* < 0.001), no ROI showed increased connectivity in controls relative to synaesthetes.

We also compared the connectivity for the sentences > scrambled sentences contrast, masking the analysis with the volume activated by perception of sentences minus the baseline in synaesthetes (voxelwise *P* < 0.001). No ROI showed increased connectivity in controls relative to synaesthetes or vice versa.

### Experiment 2: functional MRI study of word reading

The aim of this experiment was to delineate the set of regions involved in word reading, in order to study its commonalities with the TTS network. Participants were presented with an alternation of short visual blocks of words, faces, houses, tools and numbers, while they had to detect an oddball stimulus (the printed string, ‘######’).

#### Behavioural performance

There was no significant difference between groups on the detection task for the hit rate [98.4% in synaesthetes versus 93.4% in controls, *t* = 0.85, *P* = 0.4, 95% CI (−2.7958 to 6.7958)] or for the number of false alarms [mean = 0.47 in synaesthetes versus 0.59 in controls, *t* = −0.51, *P* = 0.61, 95% CI (−0.59 to 0.35)].

#### Reading-related activations

**Figure 4:**
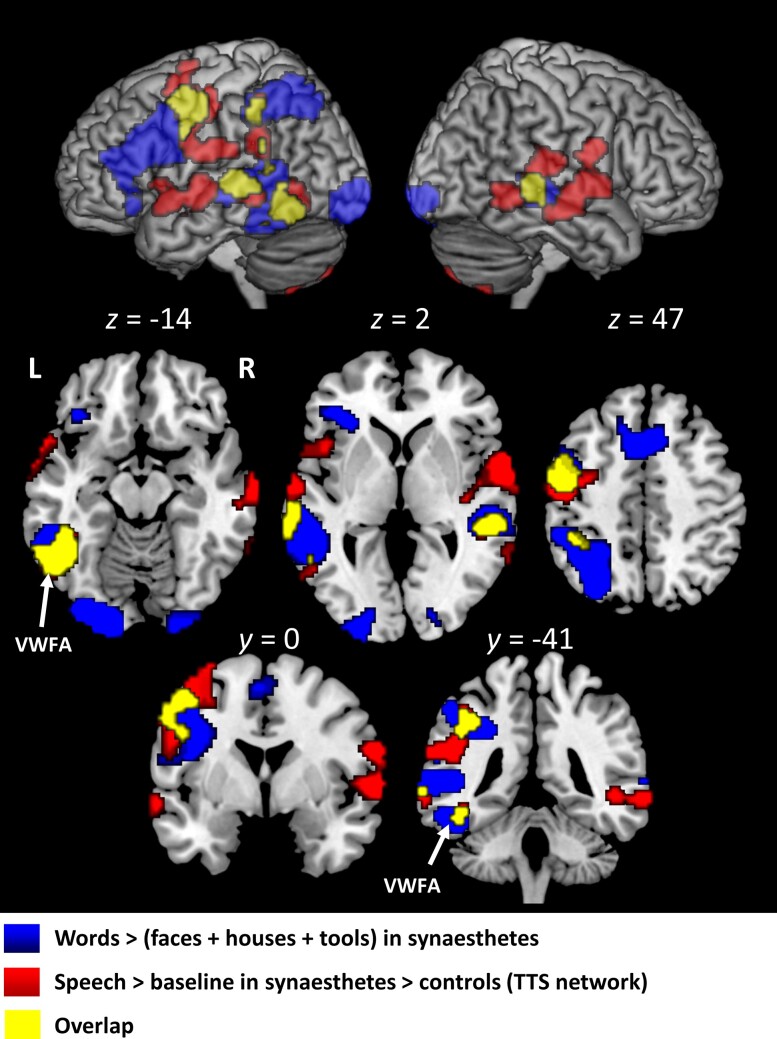
**Experiment 2.** Reading-related activation in synaesthetes, identified by contrasting words minus faces, houses and tools (blue), including the visual word form area (VWFA, white arrows). This activation overlapped largely (yellow) with the TTS network as identified in Experiment 1 (red). Abbreviations: L = left; R = right; TTS = ticker-tape synaesthesia.

To identify reading-related activations, we contrasted words minus the averaged faces, houses and tools. Across both groups, words activated a broad network of strongly left-lateralized fronto-temporo-parieto-occipital areas, including the VWFA (in all participants: MNI −50, −54, −14, *Z* > 8; in synaesthetes: MNI −50 −56 −12, *Z* = 6.78; in controls: MNI −50, −54, −17, *Z* = 6.17; Fig. 4; Supplementary Fig. 5). There was no significant difference between groups. The simple contrast of words minus baseline did not differ between groups.

#### Other category-specific activations

We identified other category-specific activations by separately contrasting faces, houses and tools, each minus the other conditions including words. We also contrasted words versus numbers. We found the usual ventral occipito-temporal mosaic (Supplementary Fig. 5) comprising the bilateral lateral occipital cortex for tools and parahippocampal place area for houses, and the right-hemispheric fusiform face area and occipital face area for faces, plus stronger activation for words than numbers in the left hemisphere. None of those contrasts differed between synaesthetes and controls.

#### Top-down activation of the visual word form area by speech

The present experiment allowed us to define the VWFA independently from Experiment 1, based on its defining preference for alphabetic stimuli. This allowed us to confirm that the top-down activation by speech observed in Experiment 1 did prevail in the visually defined VWFA. As shown in Supplementary Fig. 6, speech activated the VWFA in a vast majority of participants, and activation was significant in both groups. Note that the VWFA component of the TTS network, i.e. the point of maximal difference in speech-related activation between TTS and controls (MNI −45, −51, −10), was slightly more mesial and dorsal than the peak of the visually defined VWFA (MNI −50, −54, −17), in keeping with the functional heterogeneity of the VWFA.

#### Overlap of speech-processing and word-reading areas

There was substantial overlap between regions more activated during speech perception in synaesthetes than in controls (i.e. the TTS network) and the reading network (Fig. 4). This was true for all components of the TTS network: the left VWFA, SMG/IPS, prefrontal cortex and the bilateral lateral temporal cortex. This overlap fitted the hypothesis that, in synaesthetes, speech processing is particularly entangled with reading. Hence, we predicted that the overlap of the two systems should be larger in synaesthetes than in controls.

**Figure 5:**
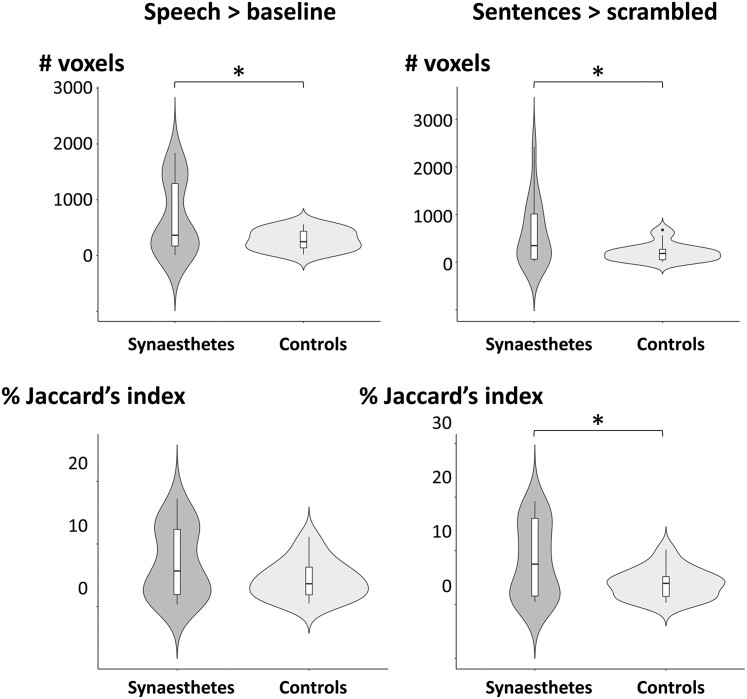
**Individual overlap of speech perception activation (Experiment 1) with reading activation (Experiment 2), thresholded at voxelwise *P* < 0.001**. Speech perception was defined by the contrast of speech > baseline (*left column*) and normal > scrambled sentences (*right column*). The overlap was defined as the number of voxels (*top row*) or Jaccard’s index (*bottom row*). *P < 0.05.

To assess this prediction, we delineated in each participant the volumes activated by speech minus baseline (Vs) and by word reading minus faces, houses and tools (Vr), at five selection thresholds ranging from voxelwise *P* < 0.05 to *P* < 0.0001. None of those networks differed in size between synaesthetes and controls, for any of the selection thresholds. We then computed, for each individual, the size of the overlap between their reading network and their speech > baseline activation (Vs ∩ Vr) (Fig. 5, left column). The overlap was significantly larger in synaesthetes than in controls, for all selection thresholds from 0.05 to 0.0001 (all *P* < 0.033). Moreover, we normalized the size of the overlap by computing Jaccard’s coefficient [(Vs ∩ Vr)/(Vs ∪ Vr)]. Jaccard’s coefficient was larger in synaesthetes only for the *P* < 0.05 threshold (*P* = 0.045). This weaker difference could be attributable to the vast number of parasitic sources of individual variability in the speech network, including all levels of processing involved in the four types of speech stimuli, ranging from acoustic and phonetic up to lexical and syntactic, and including potential influences of numbers and pseudowords.

We therefore turned to the more tightly controlled definition of speech-related activations, now using the contrast of sentences minus scrambled sentences to compute Vs. Following the same method as before, we identified the activated volume in each participant and observed that it did not differ in size between synaesthetes and controls for any of the selection thresholds. We then computed its raw and normalized overlap with the reading network (Fig. 5, right column). The size of the overlap was significantly larger in synaesthetes than in controls for all selection thresholds from 0.01 to 0.0001 (all *P* < 0.030). Jaccard’s coefficient was larger in synaesthetes for all thresholds from 0.05 to 0.0001 (all *P* < 0.045).

### Experiment 3: functional connectivity during rest

In this experiment, we studied the spontaneous fluctuation of the blood oxygenation level-dependent signal during rest in order to determine whether, even in the absence of TTS, synaesthetes would show a distinctive pattern of functional connectivity.

#### Global correlation

**Figure 6:**
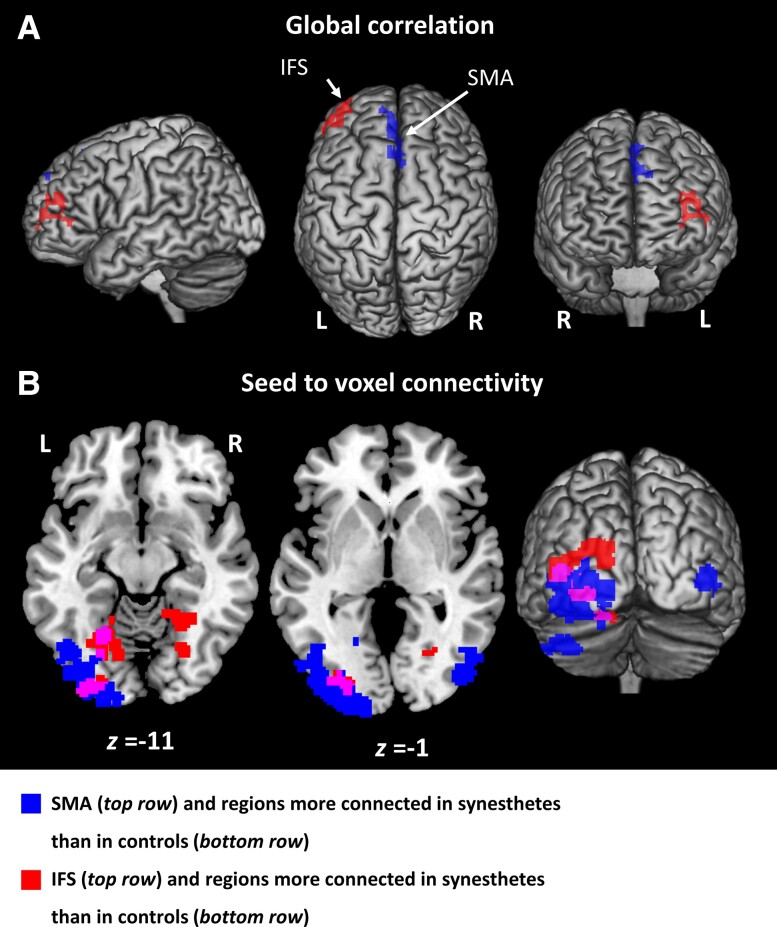
**Experiment 3.** Resting state connectivity. (**A**) Left pre-supplementary motor area (pre-SMA; blue) and inferior frontal sulcus (IFS; red) with higher global correlation in synaesthetes than in controls. (**B**) Occipital regions more correlated with the pre-SMA (blue), the IFS (red), or both (purple) in synaesthetes than in controls. Abbreviations: L = left; R = right.

We looked for brain regions more connected to the rest of the brain in synaesthetes than in controls, potentially playing a role in generating synaesthesia. To this end, we computed, for each participant, a map of global correlation, i.e. for each voxel, the average of its correlation with all the other voxels. Two regions showed higher global correlation in synaesthetes than in controls (Fig. 6A): the left anterior inferior frontal sulcus [IFS; MNI −42, 46, 20, *Z* = 3.99, *g* = 1.54, 95% CI (0.79–2.34)] and the bilateral pre-supplementary motor area [pre-SMA; MNI 6, 24, 40, *Z* = 3.95, *g* = 1.51, 95% CI (0.77–2.31)].

The left IFS is not part of the TTS network and has no obvious involvement in language and reading processes. Rather, it is involved in the access to consciousness of various perceptual representations.^32,33^ Its global over-connectivity might therefore be related to a general predisposition to synaesthesia rather than to TTS *per se*. We therefore studied whether global correlation in the IFS would be correlated with the number of associated synaesthesias (see ‘Participants' in the Supplementary material). For each synaesthete, we selected the IFS voxels with a global correlation above some threshold and computed the correlation across participants between the average global correlation of those voxels and the number of associated synaesthesias. We studied this correlation using thresholds ranging from 1% to 100% and found a tendency for synaesthetes with a higher global correlation in their IFS to have more associated synaesthesias (Supplementary Fig. 7).

Pooling synaesthetes and controls, the IFS and pre-SMA regions had a similar pattern of bilateral connectivity with the lateral and mesial prefrontal cortex and the inferior parietal lobule; the middle and inferior temporal gyri for the pre-SMA and IFS seeds, respectively; plus the occipital poles for the IFS seed (Supplementary Fig. 8). Apart from the occipital clusters, those regions correspond to the so-called executive or frontoparietal resting state network.^34^

We then assessed specifically to what areas the IFS and pre-SMA clusters were over-connected in synaesthetes (Fig. 6B). Using the IFS as seed, we found increased connectivity with the left lateral occipital cortex [MNI −42, −86, 10, *Z* = 3.96, *g* = 1.31, 95% CI (0.58–2.08)] and the bilateral collateral sulcus between about *y* = −70 and *y* = −50 [MNI 16, −46, −8, *Z* = 3.97, *g* = 0.90, 95% CI (0.20–1.62); MNI −24, −64, −10, *Z* = 3.63, *g* = 1.45, 95% CI (0.71–2.24)]. Using the pre-SMA as seed, we found bilateral occipital clusters [left: MNI −34, −96, −10, *Z* = 4.12, *g* = 1.56, 95% CI (0.82–2.37); right: MNI 46, −82, −2, *Z* = 3.92, *g* = 1.41, 95% CI (0.68–2.20)].

#### Connectivity of the ticker-tape synaesthesia network during rest

**Figure 7:**
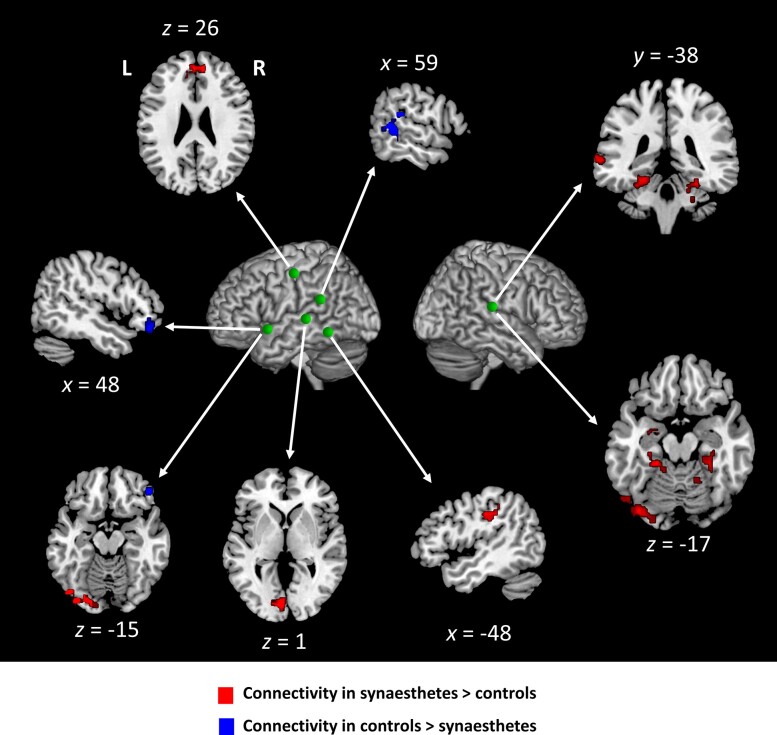
**Experiment 3.** Resting state connectivity. Regions with stronger (red) or weaker (blue) connectivity to the TTS network in synaesthetes than in controls. Green spheres represent the centres of the TTS network regions of interest showing group differences in connectivity. Abbreviations: L = left; R = right.

We then looked for regions whose rest connectivity with the nine ROIs sampling the TTS network would differ between synaesthetes and controls (*P* < 0.001 voxelwise threshold and *P* < 0.05 clusterwise FWE-corrected threshold) (Fig. 7 and Supplementary Table 2). Five of nine ROIs showed stronger connectivity in synaesthetes with left or bilateral regions: all STG ROIs (except the right anterior ROI) with the left ventrolateral and bilateral mesial occipital cortex; the right posterior STG ROI with the left temporal pole, left posterior STS/MTG and the bilateral mesial temporal; the VWFA was more connected to the bilateral postcentral sulcus; and the left precentral ROI was more connected to the bilateral mesial prefrontal cortex.

Only two ROIs showed a superiority in controls, with right-hemispheric regions: the left anterior STG with the right ventral prefrontal cortex; and the left SMG ROI with the right SMG and STS.

Finally, we reasoned that, during rest, covert speech might occasionally trigger TTS in synaesthetes and that the connectivity differences that we observed between groups might be contaminated by the corresponding activation. To assess this theory, we computed, for each individual, the average of the functional images acquired during rest and compared those images between groups. We found no difference between synaesthetes and controls, supporting the idea that group differences in connectivity reflect background predispositions and not transient phenomena related to ongoing TTS.

## Discussion

### Summary and general parsing of the findings

A group of 17 ticker-tape synaesthetes and matched controls were scanned while listening to speech (Experiment 1), viewing written words and other images (Experiment 2) and at rest (Experiment 3).

Across various analyses, synaesthetes differed from controls by showing, as a rule, more intense activation and stronger functional connectivity. Those differences, however, might not all be related specifically to TTS. Indeed, in agreement with previous studies,^35^ a majority of the participants showed one or several other synaesthesias associated with TTS.^18^ Some of the differences we found might be related to non-specific brain predispositions eventually promoting distinct types of synaesthesia.^16^

On this background, our findings can be divided into two main ensembles. On the one hand, validating our first prediction, the contrast of activation during speech perception and during baseline showed over-activation in synaesthetes in a set of left perisylvian and ventral occipito-temporal regions (the TTS network), a direct correlate of the TTS phenomenon. In agreement with our second prediction, this increased activation concerned real and pseudowords equally. Validating our third prediction, we observed a precise overlap of the TTS network with regions activated during reading and with regions involved in dyslexia, and the overlap of speech-related and reading-related areas was larger in synaesthetes than in controls. These findings bring to light the predicted link between spoken and written language processing. On the other hand, we assessed functional connectivity during rest in Experiment 3, while TTS was presumably not present. In agreement with our fourth prediction, we found over-connectivity in synaesthetes between frontal and occipital regions distinct from the TTS network. Importantly, we made sure that those differences in connectivity were not notably contaminated by the unwanted intrusion of TTS during rest in synaesthetes. We therefore propose that this pattern of increased connectivity implements a background predisposition to synaesthesia and not a transient manifestation of ongoing TTS. Finally, analyses such as psychophysiological interaction provided some indications on how these two sets of findings might be coupled.

The discussion will follow this general parsing of the results, followed by more general considerations.

### Charting the ticker-tape synaesthesia network

TTS requires the accurate mapping of input phonology to orthography, eventually allowing for vivid conscious access to the resulting written pattern. We propose that this process is implemented by the TTS network, which largely overlaps with the reading system. We will consider in turn the main components of the TTS network, analyse their involvement in the processing of speech and reading, and discuss their contribution to TTS.

#### Translating sounds into letters

The TTS network includes both anterior and posterior STG regions, with main peaks at MNI *y* = −28 and *y* = 12, respectively (Fig. 4). The STG is a complex mosaic deriving distinct representations from speech input, sometimes dissected in an anterior ‘what’ and a posterior ‘how’ pathway,^36,37^ feeding lexical and non-lexical speech processing, respectively. These two routes both contribute to TTS generation; indeed, univariate and multivariate differences between words and pseudowords did not differ between synaesthetes and controls.

#### The lexical route to ticker-tape synaesthesia

On the one hand, whenever synaesthetes listen to words with an irregular orthography, they generally see them in their mind’s eye written according to their stored spelling.^18^ The anterior STG is thought to provide the input to word recognition^38-41^ and should therefore feed the lexical component of TTS. The posterior MTG, which we found both as part of the TTS network and when decoding words from pseudowords, might be the next step in lexical TTS generation (Supplementary Fig. 3). This region is activated during lexico-semantic tasks,^42,43^ and focal lesions yield lexical deficits.^44,45^ The lexical involvement of the MTG also applies to reading, because it is consistently activated by words relative to pseudowords (for meta-analyses, see Harvey and Schnur^45^ and McNorgan *et al*.^46^). Thus the over-activated anterior STG and posterior MTG might support the lexical component of TTS, possibly in association with other lexical nodes showing no difference between groups, such as the angular gyrus (Supplementary Fig. 2).^42^

#### The non-lexical route to ticker-tape synaesthesia

On the other hand, when synaesthetes are hearing pseudowords, they see them spelled out in conformity with statistical correspondences between sounds and letters.^18^ The posterior STG, in association with the contiguous SMG, supports phonological representations for interfacing with other modalities, such as mapping to motor programmes during speech repetition,^47^ lip-reading (e.g.^48-50^) or reading and writing. There is ample evidence that the SMG is crucial for computing phoneme–grapheme correspondences. Meta-analyses show that the SMG, where we could decode words from pseudowords (Supplementary Fig. 3), is often more activated by pseudowords than by words,^51^ and per-operative or transcranial stimulation of the SMG interferes specifically with phonological but not with lexical reading.^52-55^ The posterior STG and the SMG might thus subtend the non-lexical component of TTS (Supplementary Fig. 3, bottom panel).

#### A premotor contribution to ticker-tape synaesthesia?

Finally, the left precentral component of the TTS network is commonly activated during speech production and perception,^56^ but also during reading, with stronger activation for naming than for lexical decision, for pseudowords than for words, and for longer stimuli than for shorter ones.^51,57^ It is thought to support covert speech planning, in close interaction with speech perception.^58^ One might therefore suppose that, although over-activated in synaesthetes, the premotor cortex made no causal contribution to TTS.

#### Orthography and the visual word form area

A further component of the TTS network was the VWFA, as evidence by its perfect overlap with reading-induced activation. The VWFA is best known as the entry gate for written words into the brain, but there is converging evidence that it also contributes to orthographic mental imagery, an ability possessed to some level by all literate individuals.^19,59^ Thus, brain-damaged alexic patients may lose orthographic imagery following damage to the VWFA.^60-63^ In healthy individuals, the VWFA is activated not only by strings of letters, but also during speech perception (see Fig. 1,^24,62,63^), suggesting that such top-down influences from the lexical and non-lexical routes are the source of orthographic imagery. The over-activation of the VWFA that we observed in synaesthetes would be the correlate of their exceptionally vivid and automatic imagery.

We could delineate the TTS network by using activation protocols focused on the specific type of synaesthesia we are studying. However, a majority of participants had at least one other associated synaesthesia. Using resting state data, collected while TTS was not occurring, we tried to find more general predispositions to synaesthesia, not necessarily specific to TTS.

### Fronto-occipital resting-state connectivity

During rest, the left IFS and pre-SMA had stronger global correlation in synaesthetes than in controls, specifically with the bilateral occipital cortex. The role of the prefrontal–occipital duo in controlling visual consciousness has been demonstrated in a variety of settings. According to the global neuronal workspace theory, the access of perceptual representations to consciousness results from re-entrant sharing of information between sensory regions and a supramodal network with critical prefrontal nodes.^64^ Thus, the perception threshold for masked visual stimuli is raised in patients with focal left prefrontal lesions,^32^ in multiple sclerosis patients with lesions to the prefrontal white matter and specifically to the fronto-occipital fasciculus,^65^ and in schizophrenic patients with lower anisotropy in the same fasciculus.^66^ In neglect patients, prefrontal and occipital regions are more activated whenever stimuli presented on the neglected side are perceived than when they are omitted.^67^ In healthy individuals, prefrontal activation is stronger when deficient masking allows for better subjective report of stimuli, at the exact location of our IFS cluster.^33^ Finally, prefrontal and occipital activation appears reproducibly in a meta-analysis of studies comparing activations between physically identical conscious versus unconscious stimuli.^68^

The same regions might control not only the perception of external stimuli, but also the perception of internally generated mental images (for a review, see Dijkstra *et al*.^69^). The activation of the dorsolateral and pre-SMA prefrontal cortex is modulated both by stimulus visibility during perception and by vividness during mental imagery.^70^ Moreover, closer to our finding of increased fronto-occipital correlation in synaesthetes, the functional connectivity between prefrontal and occipital areas contributes to mental imagery. Thus, dynamic causal modelling reveals a stronger excitatory influence from the prefrontal to occipital cortex during imagery than during perception.^71^ Individuals with particularly vivid mental images (hyperphantasia) show stronger fronto-occipital connectivity during rest in comparison to participants unable to generate mental images (aphantasia).^72^ In this context, the increase in fronto-occipital connectivity that we found in synaesthetes could contribute to TTS by raising to consciousness of orthographic representations that usually remain subliminal.^52,73,74^ However, those regions play no specific role in reading, and this hyperconnectivity might be expected to favour other types of synaesthesia, at least those with a visual content, consistent with our observation that most of our participants had other associated synaesthesias.^18^

### Predisposition for ticker-tape synaesthesia or dyslexia

What is the contribution to TTS of the two differences that we observed between synaesthetes and controls, namely the TTS network over-activation and the fronto-occipital over-connection? Ward^15^ proposed that any given synaesthesia results from the combination of a general ‘synaesthetic disposition’, plus innate or acquired factors steering the brain towards specific phenotypes. The general disposition surfaces, among other features, as a moderate but reproducible superiority of synaesthetes in vividness of mental imagery^75-77^ and in subjective sensory sensitivity, for instance to aversive visual glare.^78,79^ Across subjects, stronger synaesthetic disposition would result in a higher number of associated synaesthesias, a parameter that is itself correlated with several scales of imagery vividness.^77^ We assessed this hypothesis by computing the correlation across synaesthetes between the number of associated synaesthesias and the average global correlation value in the left IFS, which we identified based on its higher global correlation in synaesthetes than in controls (Supplementary Fig. 7). We found that participants with a broader set of synaesthesias tended to have higher global correlation, which should encourage further exploration. As discussed before, prefrontal regions and their connections to the occipital cortex, considering their involvement in perception and imagery, are likely to contribute to the synaesthetic disposition. In this framework, the TTS network would instantiate the functional mechanisms specific to TTS, as opposed to other synaesthesias.

The emergence of TTS results from the association of two factors: innate peculiarities in the early make-up of left-predominant areas or connections and ‘neuronal recycling’ of those systems for reading acquisition.^80^ The association of cerebral biases with the learning of reading generally results in dyslexia rather than in TTS, but we showed that common brain systems are affected in both cases (Figs 4 and 5 and Supplementary Fig. 4). Up to a point, TTS and dyslexia have opposite cerebral signatures. Considering univariate activation, the ROIs depicted in Supplementary Fig. 4 are activated below the normal level in dyslexia during reading and above the normal level in TTS during speech perception. This pattern supports the idea that peculiarities of the reading system reduce its bottom-up activation from sight in dyslexia and increase its top-down activation from speech in TTS. Disregarding the variety of dyslexias, this view would exclude the association of dyslexia with TTS, a negative prediction that remains to be assessed.

Atypical activation can be associated with atypical functional connectivity among regions of the reading network. In the case of TTS, we found increased connectivity between left temporo-parietal areas and the VWFA, with both psychophysiological interaction analyses (Fig. 3) and resting state connectivity (Fig. 7). In stark contrast, ever since the study by Horwitz *et al*.,^81^ reduced connectivity has been found repeatedly in dyslexic individuals, mostly between the VWFA and the inferior parietal lobule (IPL) and/or the lateral prefrontal cortex. This pattern prevails in adults^82^ and children,^83-85^ but also in 4- to 13-month-old infants, in whom the only predictor of family risk of dyslexia was weaker resting state connectivity of the VWFA, first and foremost with the left IPL and prefrontal frontal regions.^6^ This result provides a unique view of the cerebral predisposition for dyslexia long before reading acquisition; one may speculate that specific predispositions for TTS should involve some form of over-connectivity among the same regions.

We proposed that TTS results from the interaction of two components: a fronto-occipital system facilitating conscious access through top-down facilitation of visual imagery and an atypical reading system, in line with a hypothesis first put forward by Holm *et al*.^19^ However, it is unclear how those two components interact to generate TTS. One may note that during rest, synaesthetes showed increased cross-connectivity between both components (Fig. 7): the precentral ROI was over-connected to the pre-SMA, and both superior temporal ROIs were over-connected to the occipital cortex. Such regions of overlap might contribute to the vivid imagery of the orthographic content elicited in the reading system owing to increased top-down influence from speech areas to the VWFA.

The present results are consistent with a previous single-case study of a TTS synaesthete (individual MK), who also participated in the present study, with entirely independent data.^23^ During speech perception, individual MK showed over-activation of the core components of the TTS network, i.e. the left SMG, VWFA and precentral cortex. The present group study extended this set of regions to the lateral temporal cortex and did not replicate the mesial frontal and parietal activations observed in individual MK. The group study also confirms and generalizes the overlap of the TTS network with regions supporting word reading.

### Limitations and future directions

The subjective nature of TTS makes it difficult to define rigorous inclusion criteria, because they rely only on self-report. The self-report by our participants was supported objectively by the consistency of their introspection,^18^ by their behavioural advantage over controls^20^ (see ‘Participants' in Supplementary material) and now by their distinctive brain imaging patterns. Future tools might allow us to derive more detailed characterization of the individual features of TTS, following the example of grapheme-colour synaesthesia.^86,87^

Moreover, there is individual variability in the synaesthetic experience (location, colour, vividness, etc.). The underlying functional variability could not be elucidated in this study; in particular, we had no individual marker of subjective vividness. Mostly based on the study of grapheme-colour synaesthesia, a distinction was drawn between ‘associators’, who describe concurrent perception as internal, ‘in the mind’s eye’, and ‘projectors’, who perceive the concurrent as external, ‘in the outside world’.^88^ In the present study, only two participants could be considered as ‘projectors’ on the basis of their introspection. Exploratory comparison of those two individuals with the other (‘associator’) synaesthetes revealed no differences. As in the case of dyslexia, further pathophysiological nuances might exist depending on the involvement of different components of the reading system. For instance, does the illusory percept involve low-level visual versus more abstract graphemic representations of letters, and is this distinction reflected in the subjective features of the TTS? Are the lexical and the phonological reading routes involved to a different extent, and is this reflected in a different contribution of words and pseudowords to TTS? Clarifying those delicate issues would require careful single-case studies drawn from larger samples of participants and, possibly, time-resolved imaging techniques.^89^

Our results bring to light key functional features of TTS but provide no evidence on potential anatomical abnormalities, which might clarify the developmental causes of TTS and discriminate among general theories of synaesthesia.^21,26^ Is the over-connectivity that we identified in synaesthetes subtended by atypical anatomical connections? Do regions with an increased activity also show atypical anatomical features? Answering those questions will require the use of diffusion imaging and of morphometric techniques. A different take on the issue of causality would consist of using TMS^90,91^ to modulate components of the TTS network and study interferences with TTS, as was done with other types of synaesthesia.^92,93^

We proposed that TTS results from strong top-down activation from phonological to orthographic regions, specifically from the SMG to the VWFA. This hypothesis is consistent with the functional MRI data, but observing the information flow and the time course of phonological and orthographic codes would require time-resolved techniques (EEG or magnetoencephalography), which have been successfully applied to grapheme-colour synaesthesia (e.g. Brang *et al*.^94^ and Teichmann *et al*.^95^).

Finally, longitudinal studies in children, before, during and after reading acquisition, would be crucial for identifying synaesthetic predisposition, observing the emergence of activation patterns associated with synaesthesias involving orthography and their relationships with typical reading-related activations,^3,96^ evaluating the early incidence of TTS and its stability over time, and evaluating the relationships between TTS and dyslexia. Such studies could be set up practically as ancillary components of broader studies of reading development.

## Supplementary Material

awae114_Supplementary_Data

## Data Availability

Data are available at https://doi.org/10.5281/zenodo.8164435. No part of the study procedures or analyses was pre-registered prior to the research being conducted.
