# Supplementary material for: Subtitled speech: the neural mechanisms of ticker-tape synaesthesia
Source: Brain. 2024 Apr 15;147(7):2530–41. doi: 10.1093/brain/awae114 (PMC11224615; doi:10.1093/brain/awae114)
Supplement: awae114_Supplementary_Data [file awae114_supplementary_data.pdf]

Supplementary material

Index of supplementary material

Participants

Supplementary methods

- MRI acquisition and preprocessing
- Experiment 1: fMRI study of speech perception
- Experiment 2: fMRI study of word reading
- Experiment 3: Functional connectivity during rest

Supplementary figure 1

Supplementary figure 2

Supplementary figure 3

Supplementary figure 4

Supplementary figure 5

Supplementary figure 6

Supplementary figure 7

Supplementary figure 8

Supplementary table 1

Supplementary table 2

Supplementary references

Participants

Characteristics of synesthetes and controls

We created a short description of TTS, and recruited participants by broadcasting it through email and social networks, targeting particularly groups of students and university members, and groups devoted to synesthesia, psychology, and neuroscience. We broadcasted a short description of what TTS is, and recruited 22 ticker-tape synesthetes through email and social networks. Participants answered a detailed questionnaire about the visual and temporal characteristics of their synesthesia (see (Hauw et al., 2022) for details). They all automatically experienced TTS when listening to speech, and only a few of them could to some extent inhibit it voluntarily. Most participants had associated synesthesias (**see Table and Figure below**). Moreover, as detailed below, synesthetes enjoyed an objective behavioral advantage over controls, supporting our recruitment criteria. We recruited 22 controls matched one-to-one to synesthetes based on age, gender, education level and handedness. We excluded 5 left-handed participants in each group, resulting in 2 groups of 17 participants. All had post-secondary education; both groups comprised 4 males; the mean age was 37.6 years (range 18.6-70.8) for synesthetes and 37 years (range 19.5-70.4) for controls. All participants were native French speakers, right-handed according to the Edinburgh Inventory (Oldfield, 1971), had normal or corrected-to-normal vision and hearing, and had no history of neurological or psychiatric disorders. The research was approved by the institutional review board of the INSERM (protocol C13-41), and all participants provided informed written consent in accordance with the Declaration of Helsinki.

Behavioral differences between synesthetes and controls

The current participants were a subset of the participants in a previous study (Hauw et al., 2023). In this study, we reasoned that their enhanced orthographic mental imagery should provide TTS synesthetes with an advantage in orthographic tasks on spoken input. We designed letters counting, backward spelling, and letter shape decision tasks, all with spoken input. Those 3 tasks are relatively difficult to perform mentally, while they would be much easier with an external or internal written support. For full methodological details, please refer to the original publication (Hauw et al., 2023).

Here, we analyzed those behavioral data, restricting them only to the set of participants in the current study.

In Experiment 1 (**letters counting**), synesthetes made fewer errors (13.5% vs 26.5%; $\chi^2(1)=5.21$, $p=0.022$) and responded faster (3444 ms vs 4615 ms; $F(1,31)=13.1$, $p=0.001$) than controls. In Experiment 2 (**backward spelling**), there was a tendency for synesthetes to make fewer errors than controls (13.7% vs 20.4%; $\chi^2(1)=2.7$, $p=0.10$), but they responded more than 3 s faster (5237 ms vs 8715 ms; $F(1,30)=6.64$, $p=0.015$). In Experiment 3 (**letter shape detection**), synesthetes made fewer

errors than controls (5.8% vs 14.9%; $\chi^2(1)=6.4$, $p=0.011$) and responded faster (mean correct RT: 1379 ms vs 1536 ms; $F(1,32)=5.84$, $p=0.022$).

We thus showed that the objective behavioral advantage of synesthetes was reliably present in the current set of participants, providing further objective support to our recruitment criteria.

Note that we also predicted that synesthetes should perform worse than controls in difficult orthographic tasks on written input, due to the irrepressible intrusion of synesthetic letters induced by simultaneous spoken distractors. However, in the original study, as well as the current restricted analyses, we failed to observe such difference between groups. Thus in Experiment 4 (**interference of speech on visual lexical decision**), we found no difference between groups for error rate (27.2% vs 27.8%, $\chi^2(1)=0.06$, $p<1$) nor response times (mean correct RT: 625 ms vs 638 ms, $F(1,32)=0.05$, $p<1$). In Experiment 5 (**interference of speech on letter decision**), we found no difference between groups for error rate (8.3% vs 15.2%, $\chi^2(1)=1.73$, $p=0.19$) nor response times (mean correct RT: 591 ms vs 653 ms; $F(1,32)=1.23$, $p=0.276$).

	**N = 17**
**Space-time**	7 (41%)
**Number-space**	6 (35%)
**Sound-color**	5 (29%)
**Grapheme-color**	3 (18%)
**Digit-color**	3 (18%)
**Perfect pitch**	3 (18%)
**Ordinal-linguistic**	2 (12%)
**None**	5 (29%)

Associated synesthesias in the 17 TTS synesthetes.

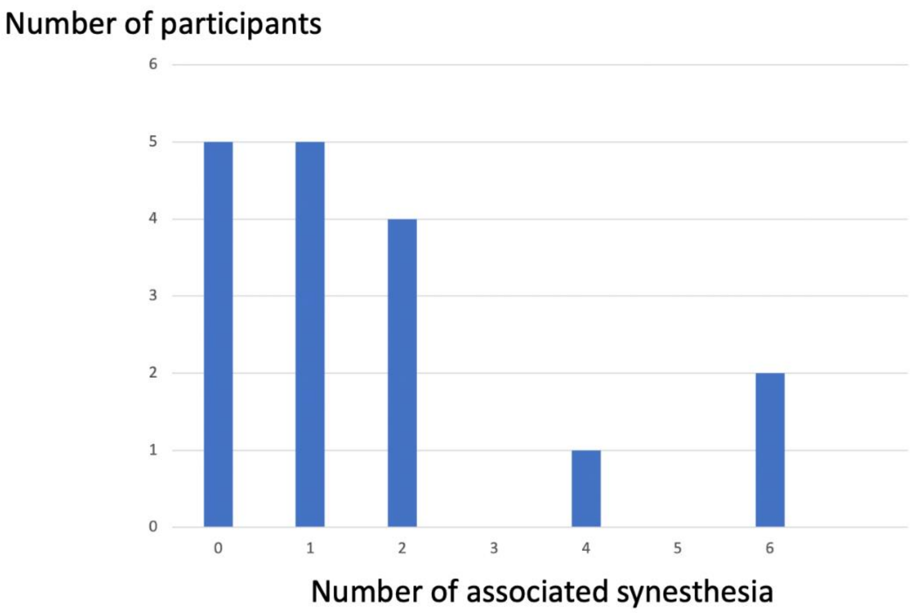

Number of associated synesthesias (TTS not included) in TTS synesthetes. Five (29%) synesthetes only had TTS, while the others had 1 to 6 associated synesthesias.

Supplementary methods

MRI acquisition and preprocessing

All images were acquired on a Siemens 3T MAGNETOM Prisma scanner. T1-weigthed anatomical images (TR/TE/flip angle = 2400 ms / 2.23 ms / 9°, voxel size = 0.7 × 0.7 × 0.7 mm) were acquired with a 64-channel receive-only head coil. For functional images, we used multi-echo fMRI sequences (Posse, 2012) : time repetition (TR) = 1660 ms; multi-time echo (TE) = 14.2, 35.39, 56.58 ms; flip angle = 74°; voxel size = 2.5 × 2.5 × 2.5 mm; 60 slices; 20-channel receive-only head coil. Cardiac and breathing frequencies were recorded using a pneumatic breathing belt and a pulse oximetry unit.

Using CAT12 (http://www.neuro.uni- jena.de/cat), anatomical volumes were segmented, normalized to the standard MNI stereotactic space and resampled to 1.5 x 1.5 x 1.5 mm. Functional images were processed as follows. Using afni_proc.py, all echoes time-series were despiked and slice-time corrected. Motion correction was computed on the first echo with the volume with the lowest motion as reference, then applied to all echoes, and finally merged using optimal combination t2smap.py (see meica.py provided with AFNI: http://afni.nimh.nih.gov/afni/). Finally, using SPM12 (Wellcome Institute of Imaging Neuroscience, London, UK) implemented in Matlab, functional time-series were co-registered to the anatomical volume, then normalized to Montreal Neurological Institute (MNI)-space (2.5mm isotropic), and spatially smoothed (5mm full-width at half-maximum [FWHM] isotropic).

Experiment 1: fMRI study of speech perception

Stimuli and procedure

There were 5 types of stimuli: spoken pseudowords, words, numbers, sentences and scrambled sentences. They were presented in a block design, with interspersed blank periods, in random order. Auditory words and pseudowords were a subset of those used in Bouhali et al. (2019). Words and pseudowords ranged from 5 to 10 letters, and from 1 to 3 syllables. Numbers ranged from 1 to 4 syllables and did not significantly differ in length from words. The sentences were consecutive fragments of the Snow-White tale. The stimuli for those first 4 conditions, which will be subsequently referred to as “the speech conditions”, were generated using the ttsreader.com speech generation software with a female voice. The stimuli of the fifth condition (scrambled sentences) were derived from the sentences using an algorithm preserving the spectral content over long time scales while removing structures at shorter timescales (www.mathworks.com/matlabcentral/fileexchange/29396-time-domain-scrambling-of-audio-signals (Ellis, Dan, 2023)). The experiment lasted 13.4 minutes. To optimize the design for multivariate pattern analysis (MVPA), we divided the experiment in 8 blocks of 1.3 minutes each, separated with 15 s silent periods. Each block consisted of 5 mini-blocks, one for each type of stimuli, separated with 3 s silent periods. Moreover the experiment started and ended with a 15 s silence. Participant had to detect the occasional target pseudoword “tatatata”, occurring

once randomly in half the mini-blocks, pronounced by the same voice as stimuli. Stimuli were presented using Psychtoolbox v3 in MATLAB (Mathworks, Natick, MA, USA).

Statistical analysis

For single-subject analyses, we defined a general linear-model (GLM), with regressors for all experimental condition (words, pseudowords, sentences, scrambled sentences, numbers), plus a regressor for targets and one for motor responses. Those regressors were created by convolving boxcar functions based on the onsets and durations of events with a canonical hemodynamic response function (HRF). The GLM also included 24 motion parameters, and regressors modeling physiological noise derived from cardiac and respiratory activity (TAPAS toolbox, https://github.com/translationalneuromodeling/tapas); Time-series were high-pass filtered (128 s cutoff). Individual contrast images of each of the 5 conditions vs baseline were smoothed (8mm FWHM) and entered in a second-level (or group-level) ANOVA with subject as random factor, group as between-subjects factor and conditions as within-subjects factor. Unless stated otherwise, the statistical threshold was set to $p<0.001$ voxel-wise, and to $p<0.05$ cluster-wise FWE-corrected. This means that on the voxels which survived the voxel-wise threshold, we applied a threshold on cluster size, which was set to $p<0.05$ after FWE-correction for multiple comparisons across the whole brain. When describing brain activation by a given contrast, we report the anatomical labels of activated regions, the coordinates of those regions' peak voxels, the Z-value associated to the contrast in those peak voxels, as well as Hedges' g effect size and its 95% confidence interval (Gerchen et al., 2021a).

Definition of individual ROIs

Based on activations in Experiment 1, we defined individual regions of interest (ROIs) for use in functional connectivity analyses. For each participant, we created 9 ROIs, defined as 4 mm-radius spheres. Those spheres were centered on the participant's voxel most activated (highest t-value) by the contrast speech > baseline within a 6 mm-radius sphere centered on the main peaks of the "TTS network", defined by the group-level contrast (speech > baseline) x (synesthetes > controls) (see Results). These 9 ROIs were used as seeds for the analysis of resting-state data. We created a second set of 9 ROIs for each subject, using the same method, except that participants' most activated voxel was identified using the contrast sentences > scrambled sentences. These ROIs were used as seeds in psychophysiological interaction (PPI) analyses.

Psychophysiological interaction (PPI) analysis

Our aim was to study condition-dependent changes in the functional connectivity between specific seeds and the rest of the brain, and to compare those changes between groups. Specifically, we compared connectivity during speech vs baseline, and during sentences vs scrambled sentences. We used the default SPM12 PPI method. For each participant, each pair of compared conditions, and each seed ROI (see above), we computed a contrast image comparing the correlation map of the seed with the rest of the brain during the two compared conditions. Those first-level images were smoothed (FWHM 8 mm) and entered in second-level ANOVAs, one for each pair of compared conditions, with subject as random factor, group as between-subjects factor and ROIs as within-subject factor. The statistical threshold was set to $p<0.001$ for the voxel-wise threshold, and to $p<0.05$ for the cluster-wise threshold FWE-corrected for multiple comparisons across the whole brain.

Multivariate pattern analysis (MVPA)

We used The Decoding Toolbox (Hebart et al., 2015), in order to decode words from each of the other conditions based on multivoxel activation patterns. We applied a leave-one-block-out cross-validation design across the 8 blocks of the experiment. Decoding was performed across the whole brain, with a 10 mm-radius searchlight. Decoding performance around each voxel was quantified as the accuracy above the 0.5 chance level. Individual accuracy maps were then smoothed (8mm FWHM) and entered in two-sample t-tests. The statistical threshold was set to $p<0.001$ for the voxel-wise threshold, and to $p<0.05$ for the cluster-wise threshold FWE-corrected for multiple comparisons across the whole brain.

Experiment 2: fMRI study of word reading

Stimuli and procedure

The stimuli consisted of printed words, Arabic numbers, pictures of houses, faces and tools, presented in a block design, with interspersed blank periods, in a random order. Words ranged from 3 to 5 letters (mean=4.8). Numbers were matched to the words in orthographic length. Houses, faces and tools were black and white pictures. Luminance and contrast did not differ across categories. Stimuli were displayed in the center of a grey circle on a black background. The experiment was divided in 8 blocks, of 1.6 minutes each, separated with a 15 s silent period. Each block consisted of 10 mini-blocks, i.e. 2 mini-blocks for each condition, separated with a 3 s silent period. Moreover the experiment started and ended with a 15 s silence. Subjects had to detect the visual target “######”, occurring once randomly in half the mini-blocks. The experiment lasted 15.8 minutes. Stimuli were presented using Psychtoolbox v3 in MATLAB (Mathworks, Natick, MA, USA).

Statistical analysis

We used the same methods as in Experiment 1, except that the factors of interest now corresponded to the 5 types of visual stimuli.

Experiment 3: Functional connectivity during rest

Procedure

During 10 minutes, participants had to fixate a central white cross on a black screen, while the room was in darkness.

Preprocessing

Images were first processed through the same general pipeline as Experiments 1 and 2. Then, applying the default settings of the Functional Connectivity Toolbox (CONN) (Whitfield-Gabrieli and Nieto-Castanon, 2012), the signal from the white matter and CSF was regressed out, and a linear detrending and a band-pass filter of 0.008–0.09 Hz were applied.

Statistical analysis

Analyses were performed with the CONN toolbox. Single-subject analyses included the same regressors for motion and physiological noise as in Experiments 1 and 2. We performed ROI-to-voxel analyses using ROIs derived from Experiment 1, and voxel-to-voxel analysis of Global Correlation. The statistical threshold was set to $p<0.005$ for the voxel-wise threshold, and to $p<0.05$ for the cluster-wise threshold FWE-corrected for multiple comparisons across the whole brain.

Supplementary figure 1

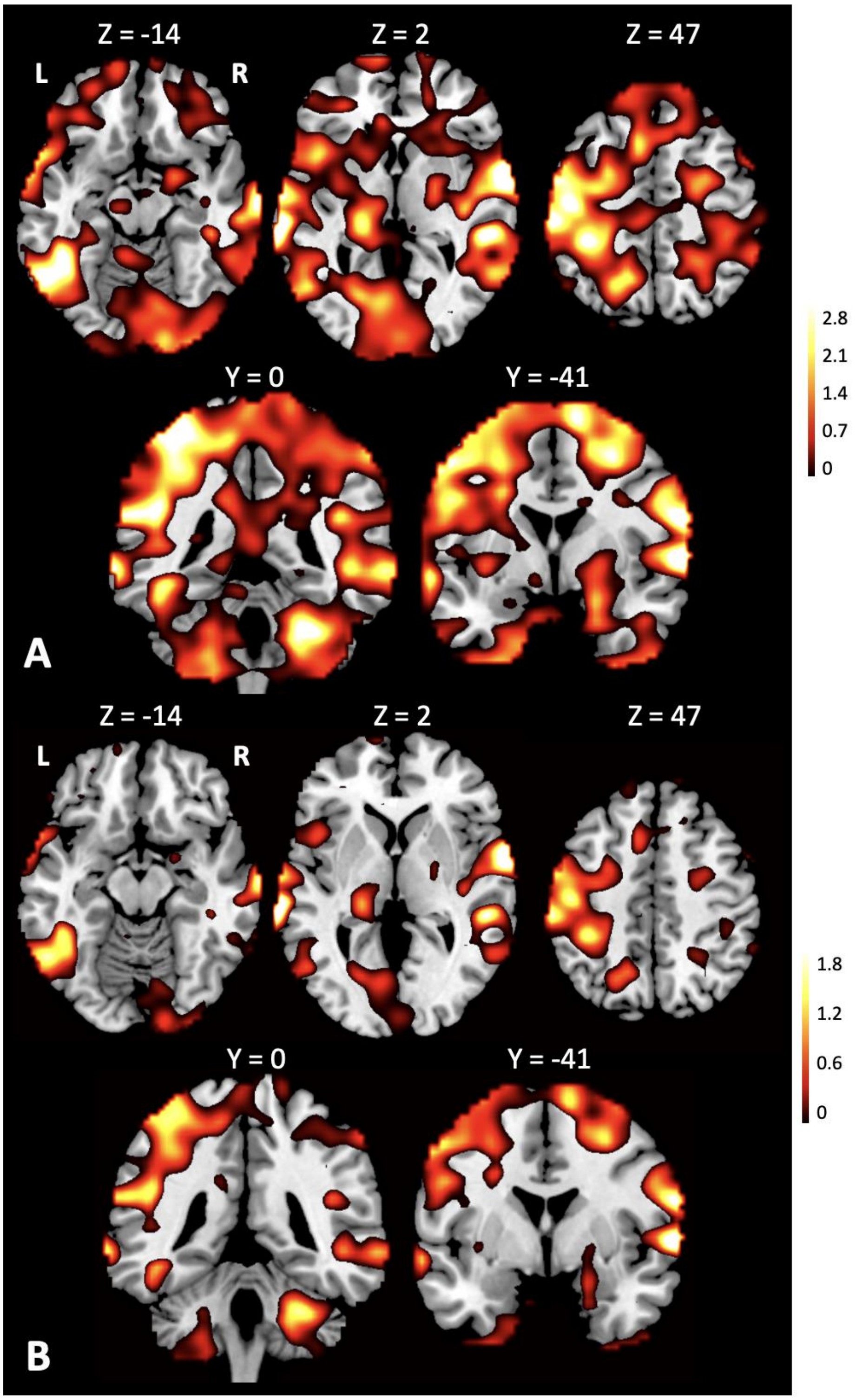

<u>Maps of Hedges' g index of effect size.</u> In Experiment 1, we compared the brain activations for Speech > Baseline in Synesthetes > Controls and thus identified a set of regions which we labelled the "TTS network". For this same contrast, we computed Hedges' g index of effect size (Gerchen et al., 2021b). (A) The effect size was higher in all parts of the TTS network. (B) The lower boundary of the 95% CI of the effect size was > 0 in all parts of the TTS network.

Supplementary figure 2

Univariate comparison between the 4 speech conditions

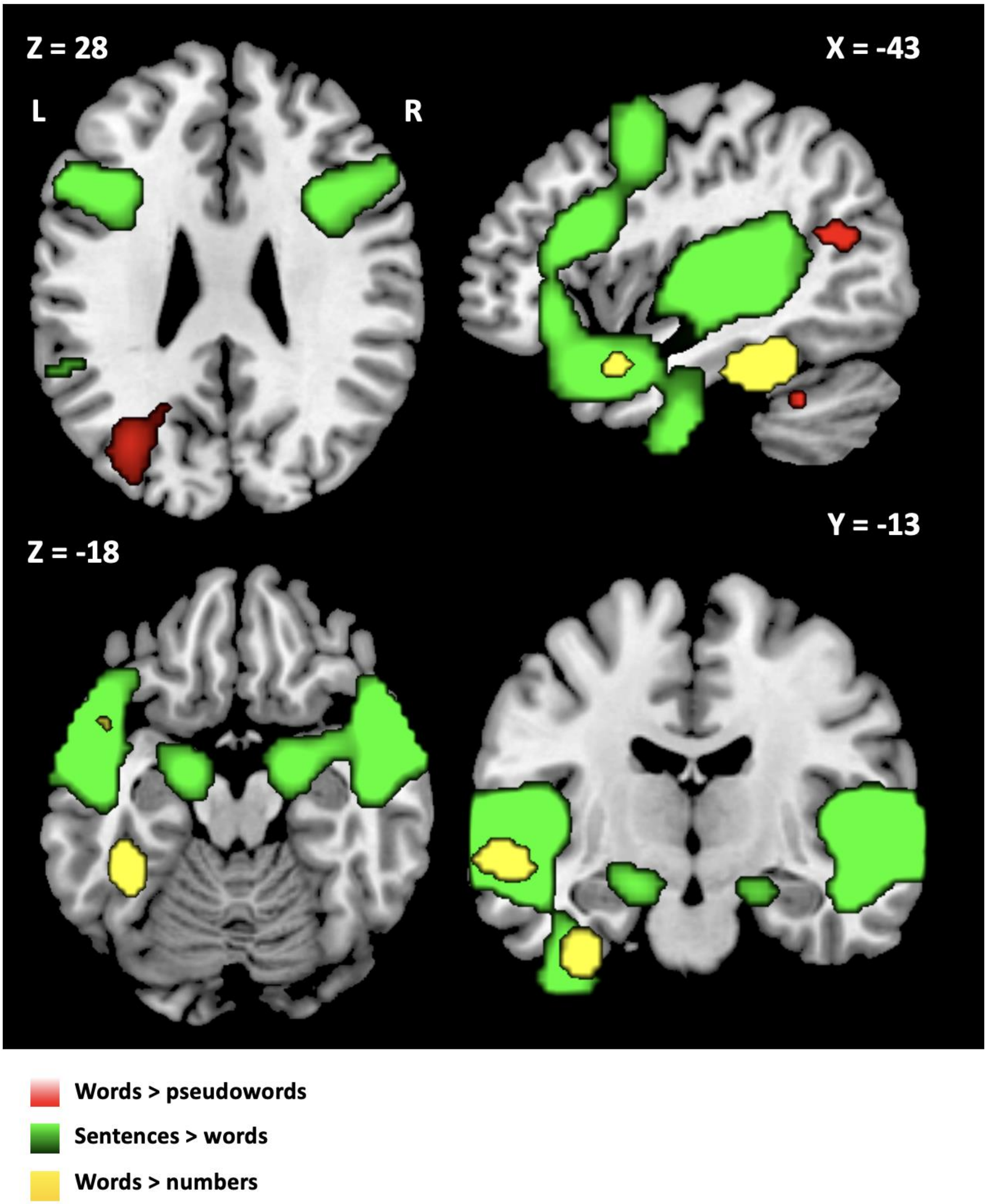

In **Experiment 1**, we compared activations induced by the 4 speech conditions (sentences, lists of words, lists of pseudowords, numbers). We computed univariate contrasts of words vs pseudowords, words vs sentences, and words vs numbers, in both directions. Averaging synesthetes and controls, we found:

1. stronger activation by words than pseudowords in the left angular gyrus (MNI -30 -71 33; Z=4.83);
2. stronger activation by sentences than words in an extensive bilateral fronto-temporal network; conversely, there was stronger activation by words than sentences, actually corresponding to stronger deactivation by sentences in the default mode network;
3. stronger activation by words than numbers in the left fusiform gyrus from about y=-50 to -5 (MNI -38 -36 -24; Z=5.04; MNI -32 -8 -37; Z=4.10) and in the left anterior STS/STG (MNI -58 -8 -7; Z=4.92).

None of those contrasts between speech stimuli differed between synesthetes and controls.

Statistical threshold: p<0.001 voxel-wise, and p<0.05 cluster-wise, FWE-corrected for multiple comparisons across the whole brain.

Supplementary figure 3

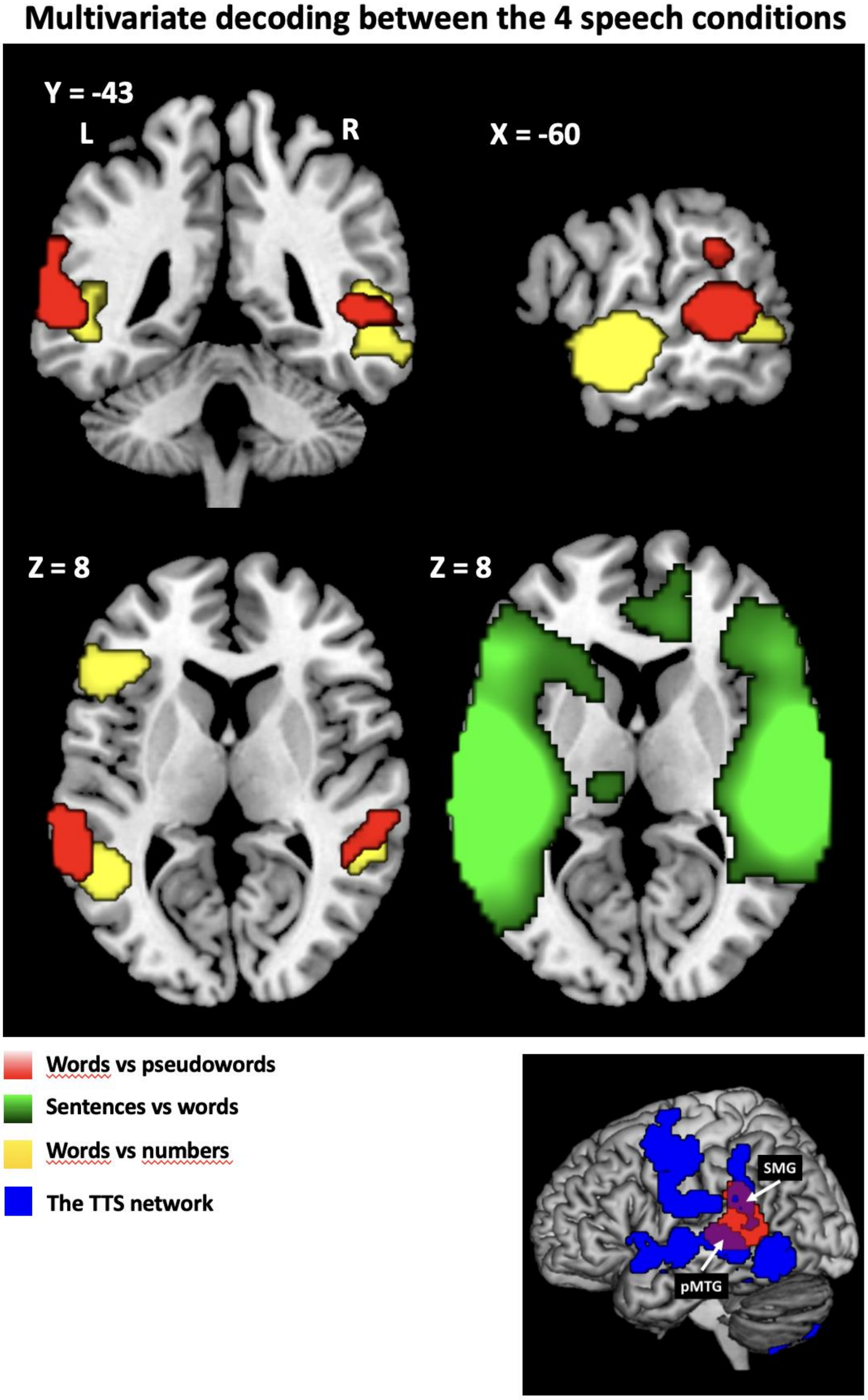

In **Experiment 1**, we also used an MVPA searchlight to decode activation by words vs each of the other speech conditions. Averaging both groups, we found:

1. the left posterior superior temporal sulcus (STS) / SMG (MNI -65 -41 6; Z=4.73) and the right STS / anterior STG (MNI 65 -24 -4, Z=4.26) distinguished words from pseudowords;
2. extensive bilateral fronto-parieto-temporal regions (MNI -55 -8 3; Z>8) distinguished words from sentences;
3. the left anterior STG (MNI -55 -8 -4; Z=5.85), the left posterior STS (MNI -48 -54 10; Z=3.94), the right angular gyrus (MNI 45 -64 28, Z=4.36), and the right posterior STS (MNI 55 -41 0, Z=4.24) distinguished words from numbers.

Like in univariate analyses, we found no difference between synesthetes and controls.

Statistical threshold: $p<0.001$ voxel-wise, and $p<0.05$ cluster-wise, FWE-corrected for multiple comparisons across the whole brain.

The bottom panel shows the overlap of the left-hemispheric region decoding words from pseudowords (red), and the posterior STG and SMG components of the TTS network (blue).

Supplementary figure 4

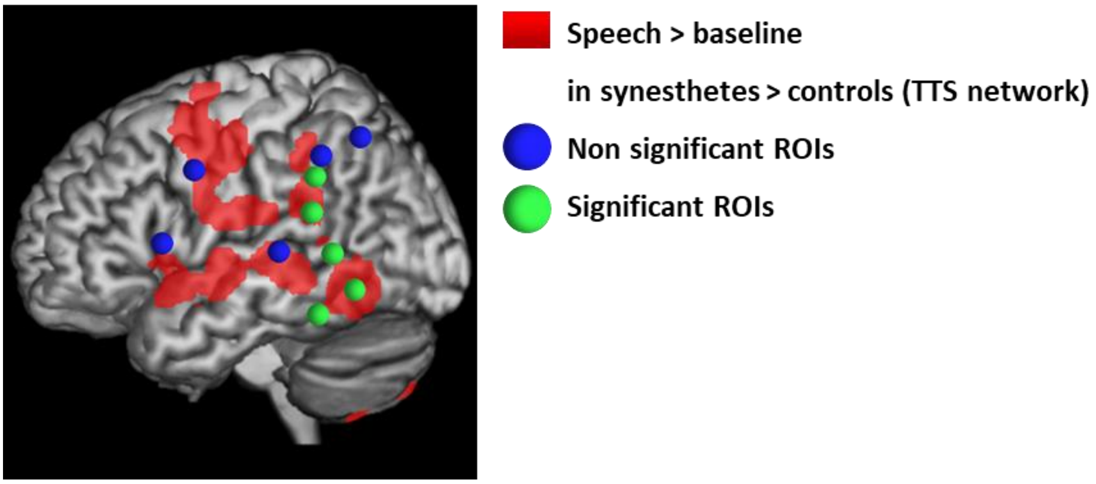

Experiment 1. Overlap of the TTS network (red) with regions of interest involved in dyslexia (centered on the spheres). Activation by speech was stronger in synesthetes than in controls in parietotemporal ROIs (green). Although not significant the other ROIs were also contiguous to the TTS network (blue).

Supplementary figure 5

Category-specific activations in all participants

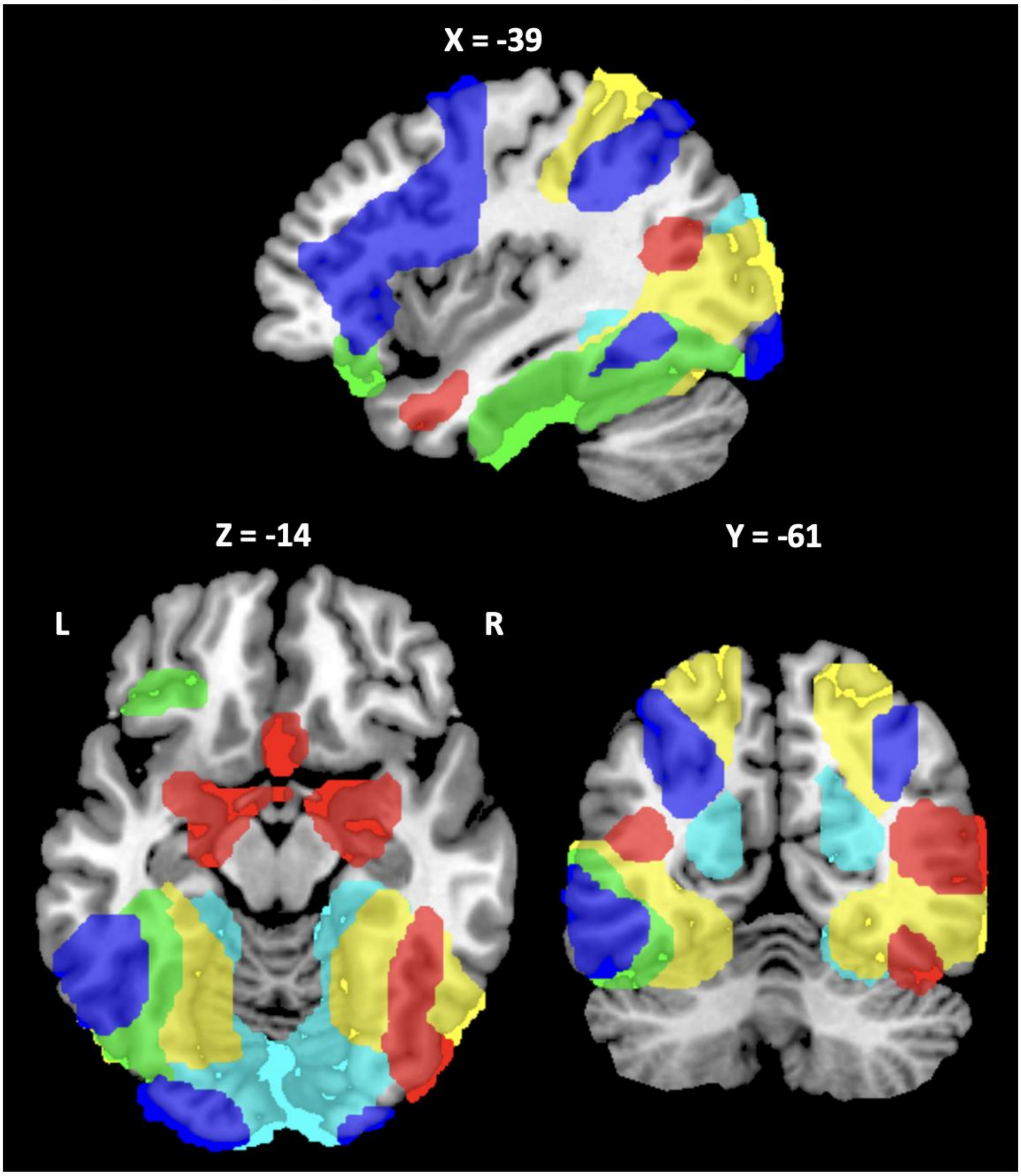

Activations in synesthetes and controls

- Words > (tools + faces + houses)
- Faces > (words + tools + houses)
- Tools > (words + faces + houses)
- Houses > (words + faces + tools)
- Words > numbers

In **Experiment 2**, we contrasted faces, houses, and tools each minus the average of the other two and words. Averaging both groups, we found the usual VOTc mosaic comprising the bilateral LOC for tools (left: MNI -40 -74 3, Z>8; right: MNI 45 -64 -4, Z>8) and PPA for houses (left: MNI -25 -48 -7, Z>8; right: MNI 28 -46 -7, Z>8), and the right-hemispheric FFA (MNI 42 -46 -22, Z>8) and OFA (MNI 50 -64 16, Z>8) for faces. We also contrasted words vs numbers in both directions. There were stronger activations for words than numbers in the left-hemisphere, along the ventral occipitotemporal cortex from about y=-90 to y=0, peaking close to the VWFA (MNI -42 -38 -22; Z=7.63), the posterior STS (MNI -55 -54 6; Z=5.71), and the IFG down to the orbitofrontal cortex (MNI -45 32 10; Z=6.69). There were no stronger activations for numbers than for words. None of those contrasts differed between synesthetes and controls. Statistical threshold: $p<0.001$ voxel-wise, and $p<0.05$ cluster-wise, FWE-corrected for multiple comparisons across the whole brain.

Supplementary figure 6

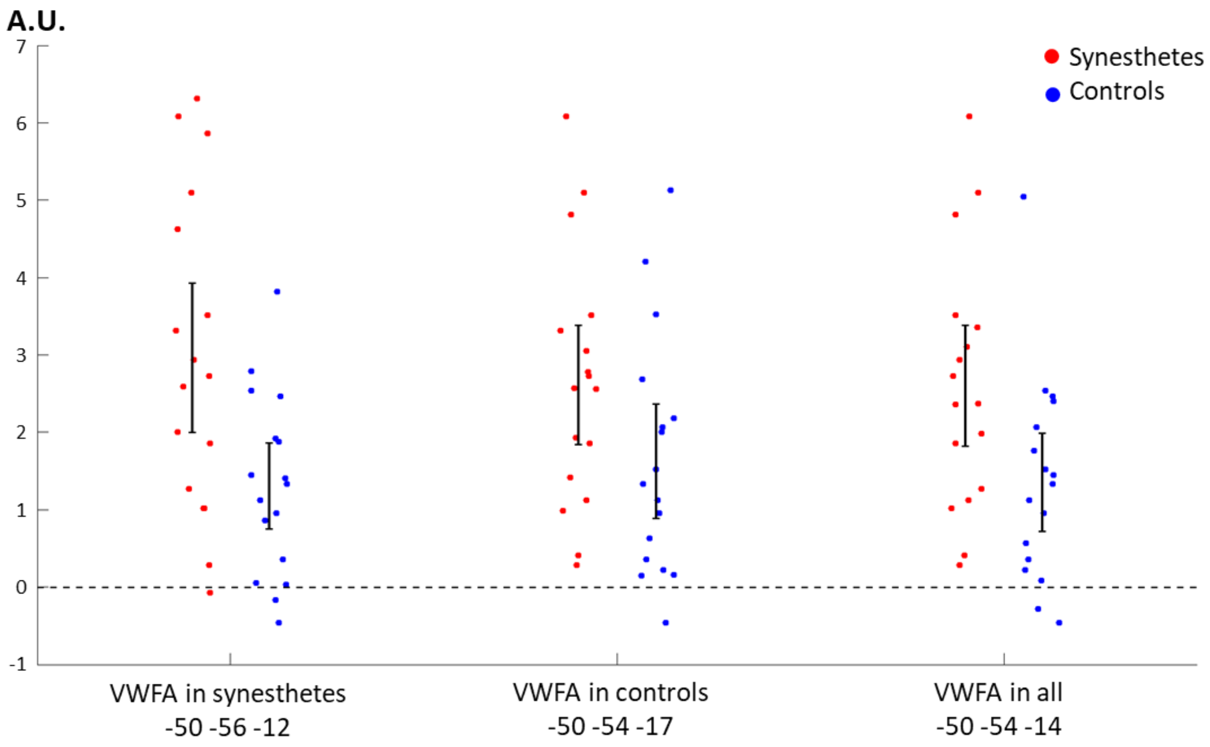

Activation of the VWFA by speech. We used Experiment 2 to identify the peak of the VWFA (separately in each group, plus in the two groups together), based on the fundamental criterion of category selectivity, using the contrast of words > the average of houses, tools and faces. In each participant, we identified the voxel maximally activated by the same visual contrast of words > the average of houses, tools and faces in Experiment 2 within 6 mm spheres centered on those group-level peaks. In this voxel, we looked for each participant at the activation by the contrast of speech > baseline in Experiment 1. Individual activation values are plotted in red for synesthetes, and in blue for controls, showing that speech activated the VWFA in a large majority of participants. Black bars represent confidence intervals. The 95% confidence intervals never included 0, demonstrating significant activation by speech in both groups at the peak of the VWFA.

The same conclusion was reached studying individual activations at the group-level coordinates of the VWFA, rather than at the individual maxima as shown in the current Figure (Z>8 at the three depicted peaks).

Supplementary figure 7

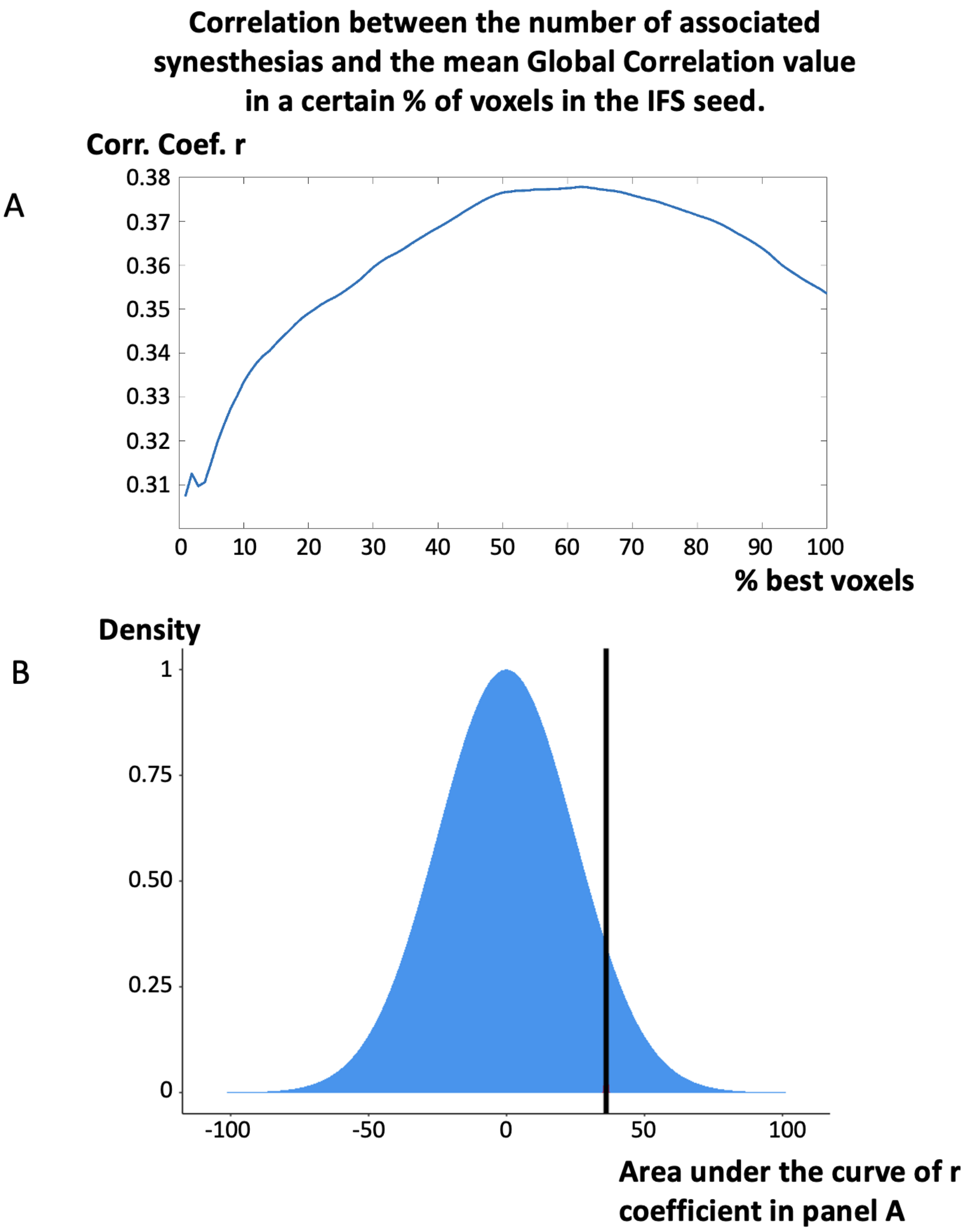

Experiment 3: In each synesthete, we selected the voxels with the highest Global Correlation (GC) from the left IFS region with higher GC in synesthetes than in controls.

(A) We used 100 different thresholds for selecting the voxels (the 1% to 100% best voxels, with a 1% step), and computed at each threshold the correlation between the mean GC of those voxels and the

number of associated synesthesia. All R values were positive (all R>0.30), which would be unexpected in the absence of an actual positive correlation.

(B) To statistically assess this observation, we performed 10.000 permutations of the number of synesthesias, and for each permutation computed the equivalent of the curve in panel A, and computed the area under this curve. The plot represents the distribution of the 10000 values of this area. We compared the original area (black vertical line) to the permuted distribution (blue) with a Bayesian Crawford t-test (Crawford and Garthwaite, 2007), and found that it was larger than 93% of the permuted values (Z=1.47, p=0.071, 95% CI=0.07-0.08).

Supplementary figure 8

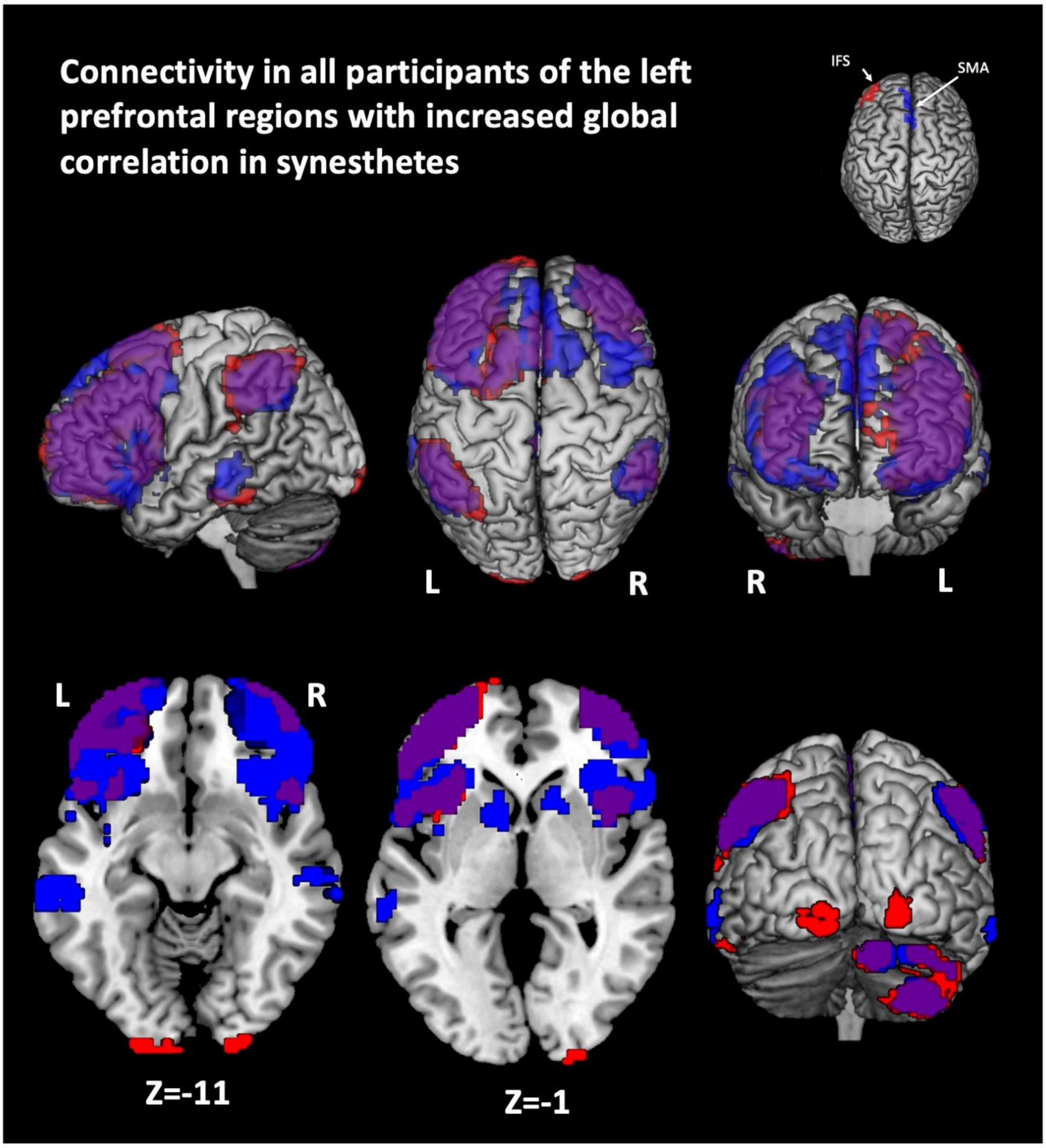

In **Experiment 3**, we looked at the connectivity of the IFS (red) and pre-SMA (blue) during rest. The seeds were identified by virtue of their stronger Global Correlation in synesthetes than in controls. The top right insert shows the location of the seeds. Averaging both groups, those two seeds had similar patterns of bilateral connectivity with the lateral (left: MNI -34 56 12, Z>8; right: MNI 38 58 10, Z=7.53) and mesial (MNI 2 34 40, Z>8) prefrontal cortex and the inferior parietal lobule (left: MNI -50 -54 40, Z=7.12; right: MNI 52 -48 40, Z=6.91), the middle (left: MNI -58 -26 -18, Z=5.24; right: MNI 62 -24 -14,

Z=4.43) and inferior temporal gyri for the pre-SMA and IFS seeds respectively, plus the occipital poles for the IFS seed (left: -12 -104 -14, Z=4.18; right: MNI 22 -104 -2, Z=4.04). For regions showing connectivity with both seeds, the reported coordinates correspond to the connectivity with the pre-SMA.

Statistical threshold: $p<0.005$ voxel-wise, and $p<0.05$ cluster-wise, FWE-corrected for multiple comparisons across the whole brain.

Supplementary table 1

MNI coordinates			
X	Y	Z	
**-44.5**	-46	-18.5	Fusiform gyrus
**-46**	-47	43	Inferior parietal lobule
**-48.8**	15.2	8.8	Inferior frontal gyrus
**-50**	-60	-9	Inferior temporal gyrus
**-52**	-43	21	Post. superior temporal gyrus
**-50**	-30.5	5	Superior temporal gyrus
**-54.5**	-51.5	5	Middle temporal gyrus
**-49.2**	-44.4	34.8	Supramarginal gyrus
**-42**	4	36	Precentral gyrus
**-34**	-62	50	Superior parietal lobule

Coordinates of the regions of interest involved in dyslexia, resulting from 4 meta-analyses compiled in Feng et al. (2020).

Supplementary table 2

	Synesthetes > Controls			Controls > Synesthetes		
Seed	**REGIONS**	**MNI**	**Z**	**REGIONS**	**MNI**	**Z**
L posterior STG	L mesial occipital	-4 -82 0	4.18	-	-	-
L anterior STG	L mesial occipital	-12 -106 10	4.48	R ventral PFC	48 38 -18	4.02
	L ventrolateral occipital	-38 -86 -18	3.72			
	R mesial occipital	2 -88 20	3.51			
R posterior STG	L temporal pole	-24 -2 -22	4.54	-	-	-
	L posterior STS / MTG	-70 -32 0	4.40			
	L mesial temporal	-22 -36 -18	4.19			
	L ventrolateral occipital	-40 -82 -14	4.16			
	R mesial temporal	-30 -38 -20	4.04			
VWFA	L postcentral suclus	-42 -22 36	3.82	-	-	-
	R postcentral suclus	66 -12 36	3.57			
L SMG	-	-	-	R SMG / STS	70 -34 20	3.85
L precentral	Bilateral mesial PFC	8 46 26	3.93	-	-	-

Experiment 3. Regions with different connectivity to the TTS network in synesthetes and in controls. L: Left; R: Right; VWFA: visual word form area; SMG: supra-marginal gyrus; STG: superior temporal gyrus; STS: superior temporal sulcus; PFC: pre-frontal cortex.

Voxelwise threshold $p<0.005$, clusterwise threshold $p<0.05$ FWE-corrected over the whole brain.

Supplementary references

Bouhali, F., Bezagu, Z., Dehaene, S., Cohen, L., 2019. A mesial-to-lateral dissociation for orthographic processing in the visual cortex. Proc Natl Acad Sci U S A 116, 21936–21946. https://doi.org/10.1073/pnas.1904184116

Crawford, J.R., Garthwaite, P.H., 2007. Comparison of a single case to a control or normative sample in neuropsychology: Development of a Bayesian approach. Cognitive Neuropsychology 24, 343–372. https://doi.org/10.1080/02643290701290146

Ellis, Dan, 2023. Time-domain scrambling of audio signals (https://www.mathworks.com/matlabcentral/fileexchange/29396-time-domain-scrambling-of-audio-signals), MATLAB Central File Exchange.

Feng, X., Altarelli, I., Monzalvo, K., Ding, G., Ramus, F., Shu, H., Dehaene, S., Meng, X., Dehaene-Lambertz, G., 2020. A universal reading network and its modulation by writing system and reading ability in French and Chinese children. eLife 9, e54591. https://doi.org/10.7554/eLife.54591

Gerchen, M.F., Kirsch, P., Feld, G.B., 2021a. BRAIN-WIDE inferiority and equivalence tests in FMRI group analyses: Selected applications. Human Brain Mapping 42, 5803–5813. https://doi.org/10.1002/hbm.25664

Gerchen, M.F., Kirsch, P., Feld, G.B., 2021b. Brain-wide inferiority and equivalence tests in fMRI group analyses: Selected applications. Human Brain Mapping 42, 5803–5813. https://doi.org/10.1002/hbm.25664

Hauw, F., El Soudany, M., Cohen, L., 2023. The advantage of being a synesthete: The behavioral benefits of ticker-tape synesthesia. Cortex 168, 226–234. https://doi.org/10.1016/j.cortex.2023.08.011

Hauw, F., El Soudany, M., Cohen, L., 2022. Subtitled speech: Phenomenology of tickertape synesthesia. Cortex S0010945222003203. https://doi.org/10.1016/j.cortex.2022.11.005

Hebart, M.N., Görgen, K., Haynes, J.-D., 2015. The Decoding Toolbox (TDT): a versatile software package for multivariate analyses of functional imaging data. Front. Neuroinform. 8. https://doi.org/10.3389/fninf.2014.00088

Oldfield, R.C., 1971. The assessment and analysis of handedness: the Edinburgh inventory. Neuropsychologia 9, 97–113. https://doi.org/10.1016/0028-3932(71)90067-4

Posse, S., 2012. Multi-echo acquisition. NeuroImage 62, 665–671. https://doi.org/10.1016/j.neuroimage.2011.10.057

Whitfield-Gabrieli, S., Nieto-Castanon, A., 2012. *Conn* : A Functional Connectivity Toolbox for Correlated and Anticorrelated Brain Networks. Brain Connectivity 2, 125–141. https://doi.org/10.1089/brain.2012.0073
